# Mediterranean Diet, Semen Quality, and Medically Assisted Reproductive Outcomes in the Male Population: A Systematic Review and Meta-Analysis

**DOI:** 10.1016/j.advnut.2025.100454

**Published:** 2025-05-24

**Authors:** Rashmi Agarwal, Jordi Salas-Salvadó, Estefanía Davila-Cordova, Sangeetha Shyam, María Fernández de la Puente, Maite Pérez Azurmendi, Nancy Babio, Albert Salas-Huetos

**Affiliations:** 1 Department of Biochemistry and Biotechnology, Universitat Rovira i Virgili, Human Nutrition Unit, Grup Alimentació, Nutrició, Desenvolupament i Salut Mental (ANUT-DSM), Reus, Spain; 2 Institute of Health Research Pere Virgili (IISPV), Reus, Spain; 3 CIBER Physiopathology of Obesity and Nutrition, Instituto de Salud Carlos III, Madrid, Spain; 4 Department of Basic Medical Sciences, Universitat Rovira i Virgili, Unit of Preventive Medicine and Biostatistics, Grup Alimentació, Nutrició, Desenvolupament i Salut Mental (ANUT-DSM), Reus, Spain; 5 Department of Nutrition, Harvard T.H. Chan School of Public Health, Boston, MA, United States

**Keywords:** male infertility, Mediterranean diet, sperm quality, medically assisted reproduction, systematic review, meta-analysis, fertility, dietary pattern, testicular function

## Abstract

Environmental pollution, sedentary lifestyles, and unhealthy dietary patterns have been hypothesized as the main modifiable factors of human semen quality decline. The study aimed to assess the associations between an a priori-defined Mediterranean diet (MedDiet) adherence and semen quality parameters or medically assisted reproductive (MAR) outcomes in males. A systematic review was conducted with studies from PubMed, Embase, or Scopus databases until October 2024. A priori-defined MedDiet adherence was considered as exposure and conventional semen quality parameters as the primary outcomes. Secondary outcomes included MAR outcomes, reproductive hormone concentrations, and sperm DNA fragmentation. A quality assessment was performed using the NHLBI tool. Meta-analysis was conducted following Cochrane guidelines. A subgroup analysis was done for healthy participants and those from fertility clinics separately. A sensitivity analysis was performed to check the influential studies. A qualitative analysis was performed on 11 eligible articles (*n* = 2558 individuals). Data from 9 observational studies showed a positive association between the adherence to MedDiet and semen volume (1/9), sperm concentration (5/9 studies), count (5/9), total motility (5/9), progressive motility (4/9), vitality (1/9), normal morphology (2/9), or follicular stimulating hormone (1/9). Among these, the links between MedDiet adherence and MAR outcomes were prospectively explored only in 1 study, which reported no association. A total of 8 studies were eligible for meta-analysis (*n* = 1835 individuals). Total MedDiet adherence showed a significant positive association with sperm count (24.37 M spz.; 1.30–47.44; *I*^2^ = 89%), total motility (8.81%; 2.26–15.37; *I*^2^ = 88%), progressive motility (7.49%; 1.47–13.50; *I*^2^ = 86%), and normal morphology (1.02%; 0.21–1.82; *I*^2^ = 77%). Evidence from 2 randomized clinical trial evaluating the effect of MedDiet on semen parameters aligns with the primary results. Evidence from observational studies and clinical trials shows potential benefit of adhering to a MedDiet in terms of seminal quality parameters, but not fertility outcomes.

This study was registered at PROSPERO as CRD42024584003.

**Statement of Significance**

To our knowledge, this systematic review and meta-analysis is the most updated and first article with the specific association of a priori Mediterranean diet on the male semen quality and medically assisted reproductive outcomes, including latest randomized clinical trials and also with meta-analyzed data for observational studies.

## Introduction

Infertility is a disease of the male or female reproductive system defined by the failure to achieve a pregnancy after 12 mo or more of unprotected intercourse [1]. This condition is considered an important public health problem affecting families and countries around the world. A total of 186 million individuals and 48 million couples are affected by infertility according to recent WHO data [2]. Among the 15%–20% of couples with infertility at reproductive age, ∼50% of the cases may be explained by male-related factors [3]. The prevalence of infertility has increased in the last decades [4], and has important social and emotional consequences for the couple and family.

Male infertility can be a consequence of some conditions and diseases, such as infection, cystic fibrosis, hyperprolactinemia, hypogonadotropic hypogonadism, disorders of ciliary function, posttesticular impairment, testicular deficiency, and obstruction in reproductive organs such as epididymis, ejaculatory duct, among others [5]. However, the increase in male infertility in the last decade can only be explained by environmental and lifestyle changes. Physical activity, obesity, smoking, alcohol intake, sleep cycle, work-life balance, pollution, endocrine disrupting chemicals, and diet are recognized modifiable risk factors of infertility [5].

Diet is considered one of the more studied risk factors because several nutrients such as omega-3 fatty acids, vitamins (vitamin D and folate), and some antioxidants (β-carotene, zinc, selenium, vitamin E, vitamin C, cryptoxanthin, and lycopene), are essential for optimal spermatogenesis and fertility [6]. Different food groups and dietary patterns containing high amounts of the aforementioned nutrients have also been related to different sperm parameters and fertility. Higher adherence to healthy diets like the Mediterranean diet (MedDiet) or Prudent dietary pattern, which is characterized by low in sugar-sweetened beverages and sugar, red and processed meat, and rich in nuts, fruits, vegetables, legumes, and whole grains, has been frequently related to better semen quality parameters [7]. In contrast, adherence to unhealthy dietary patterns, such as the “Western diet,” lower in plant-based food and fiber and rich in refined cereals and sugar, red meat, ultraprocessed food, and dairy was related to lower semen quality parameters and fertility rates [8,9]. The “Western diet” promotes weight gain, whereas the MedDiet supports a healthy weight, highlighting the key role of dietary patterns in body weight regulation [10]. Moreover, adiposity is a major critical factor that negatively affects male fertility [11]. A recent meta-analysis informed that both underweight or overweight lead to hormonal imbalances, which impair sperm production and quality [12].

A few cross-sectional and longitudinal studies have demonstrated that those participants with high adherence to the MedDiet have better semen quality parameters than those who follow unhealthy dietary patterns. Two recent systematic review and meta-analyses (SRMA) have summarized these data without including data from clinical trials. The first meta-analysis published by Cao et al. [13] included 6 studies evaluating the associations between adherence to healthy dietary patterns (including MedDiet) and semen quality parameters; these 6 studies were meta-analyzed. The second SRMA by Muffone et al. [14] specifically focused on the MedDiet analyzing the association between adherence to this dietary pattern and several fertility outcomes in males and females. However, only 3 studies were included in the meta-analysis evaluating the association between MedDiet and semen quality parameters as outcomes. More recently, Piera-Jordan et al. [15] reviewed literature relating to the association between MedDiet or some key food typical of MedDiet and seminal quality outcomes, including 7 articles that specifically focused on MedDiet, without meta-analyzing the data. In their systematic review, Piera-Jordan et al. [15] included studies published until 2022. As several articles have been published since 2022, it is important to update the existing evidence. Therefore, this study aimed to conduct an SRMA including all the studies evaluating the effect (or associations) of a priori MedDiet on semen quality, and medically assisted reproductive (MAR) outcomes in the male population. This represents the last updated summary of the scientific evidence on this topic.

## Methods

### Registration

This SRMA was conducted according to the guidelines of PRISMA [16,17] and prospectively registered in the International Prospective Register PROSPERO (https://www.crd.york.ac.uk/prospero/) with the registration ID CRD42024584003.

### Search strategies and databases

The search was performed using 3 predefined databases: *1*) PubMed (https://pubmed.ncbi.nlm.nih.gov/), *2*) Embase (https://www.embase.com/landing?status=grey), and 3) Scopus (https://www-scopus-com.sabidi.urv.cat/search/form.uri?display=basic#basic). Two authors (RA and ED-C) performed the search using a specific set of keywords in these databases, focusing on MedDiet as exposure and several outcomes related to semen quality parameters, infertility, or pregnancy outcomes, based on the titles and abstracts of the papers (Supplemental Table 1).

### Study eligibility criteria and selection

Inclusion and exclusion criteria for the study selection have been defined based on the Population, Exposure, Comparison, Outcome, Study system (Supplemental Table 2).

Studies were included if they: *1*) were conducted in human adult healthy males or males attending fertility clinics aged between 18 and 80 y, *2*) reported a priori MedDiet exposure, *3*) reported at least 1 of the following primary outcomes: semen volume, semen pH, sperm concentration, count, total motility, progressive motility, nonprogressive motility, vitality, viability, or normal morphology measured using the WHO parameters or secondary outcomes: reproductive hormone concentrations, sperm DNA fragmentation, other MAR outcomes (fertilization, implantation, clinical pregnancy, and live birth rate), and *4*) were observational case-control, cross-sectional, prospective studies, or randomized clinical trials (RCTs).

The following studies were excluded for the present SRMA: *1*) in vitro/cell culture, in silico, and animal model studies, *2*) studies including only females or studies with males aged <18 y, *3*) studies including males with azoospermia, alcohol abuse, severe chronic diseases such as cancer, *4*) studies with small sample size (<20 participants), *5*) studies with a posteriori definition of MedDiet, *6*) reviews, meta-analysis, case reports, commentary articles, and, *7*) publications in languages other than English.

The search was replicated by 2 authors (RA and ED-C) independently, to ensure reproducibility of data. Study selection was performed in 2 steps. In the 1st step, all recovered articles were screened for title, abstract, and full-text for eligibility using Rayyan software (https://www.rayyan.ai/) independently by 2 researchers (RA and ED-C), and 2 other authors (AS-H and SS) solved any conflicts. Articles found eligible for full-text screening were again independently screened to determine their eligibility, as described above.

### Data extraction

Extraction of data was performed for each of the studies included and the following information was gathered: journal name, article title, author name, year of publication, sample size, and the description of the population, country of origin of the participants, age of the population, study design, MedDiet score used [e.g., Trichopoulou MedDiet (TMD) [18], Panagiotakos MedDiet (PMD) [19], Alternate MedDiet (AMD) [20], relative MedDiet score (rMED) [21], MedDiet Adherence Screener (MEDAS) [22]] (Supplemental Table 3), seminogram parameters measured (e.g., semen volume and pH, sperm concentration, count, motility, vitality, viability, and morphology), peripheral reproductive hormones, sperm DNA fragmentation, and reproductive outcomes (e.g., fertilization, implantation, clinical pregnancy, and live birth rate). Information about relevant confounders [age, smoking status, physical activity, state and trait anxiety, total energy intake, educational level, individual income level, and family subfertility history, geographical location, body weight, BMI, type of participants (healthy compared with those recruited from fertility clinics), abstinence time before semen sample collection, reproductive organ disease history, presence of varicocele, ethnicity/race, and lipid concentrations] was also recorded. Data were extracted by 2 independent researchers (RA and ED-C) and double-checked for errors by other 2 authors (AS-H and SS).

### Quality assessment

The papers included after the full-text screening were considered for quality assessment by using the tool developed by NHLBI (https://www.nhlbi.nih.gov/health-topics/study-quality-assessment-tools). The NHLBI tool was selected because it is well-suited for observational studies, which made up most of our included studies. The study quality assessment was performed by a set of 14 questions specific for intervention studies and for observational cohort and cross-sectional studies. Two independent researchers (RA and ED-C) conducted the quality analysis, and the studies were classified as Good (10–14), Fair (4–9), or Poor (<4). Any conflicts were solved by 2 other authors (AS-H and SS).

### Statistical analysis

Meta-analyses were conducted only for 8 observational studies, which had the same exposure and outcomes, using the meta package for R 4.4.2 statistical software and Review Manager 5.4 following Cochrane guidelines [23]. For each study, the mean and SD were extracted to calculate the summary mean differences (MD) and 95% confidence intervals. If the original studies have reported the statistical values as, median, SEM, or interquartile range, then data were recalculated to mean and SD. Values were obtained by 2 authors (RA and ED-C) and cross-checked by 2 other authors (AS-H and SS) for discrepancies. Different meta-analyses were conducted taking into account the exposure: *1*) for all studies (total MedDiet), and *2*) for studies using the same score to determine MedDiet adherence (TMD, PMD, and AMD). We conducted pooled estimates both separately for each MedDiet adherence tool and combined across all tools to provide a comprehensive synthesis of the available evidence. As Hutchins-Wiese et al. [24] reported, the different MedDiet adherence tools used has only modest concordance between them and therefore, pooled estimates of the papers using each MedDiet adherence tool separately provide also estimates of dietary patterns that are defined identically. Random- or fixed-effects models were selected based on the number of studies included in the meta-analysis. A random-effects model was applied when the meta-analysis included >5 studies, whereas a fixed-effect model was used when fewer than 5 studies were included [25]. The results of the RCTs were not meta-analyzed because of the large difference in intervention duration between the studies. The statistical significance level was set at *P* < 0.05 (2-tailed). We utilized the χ^2^ tests and *I*^2^ index to assess heterogeneity among studies with the significance threshold set at *P* < 0.1. *I*^2^ values <50% were classified as moderate heterogeneity, values between 50% and 74% as substantial heterogeneity, and values of 75% or higher as considerable heterogeneity. The publication bias was assessed qualitatively by analyzing the presence of asymmetry in the funnel plot. Sensitivity analysis was performed by excluding one article at a time for only the total MedDiet effect on semen parameters. We considered an influential study on the associations when it changed the significance, direction or magnitude (by >20%) of the pooled MD, or changed the magnitude of the heterogeneity (e.g., considerable heterogeneity to substantial). Subgroup meta-analysis was performed separately for healthy participants or those recruited from fertility clinics.

## Results

### Article selection

**FIGURE 1:**
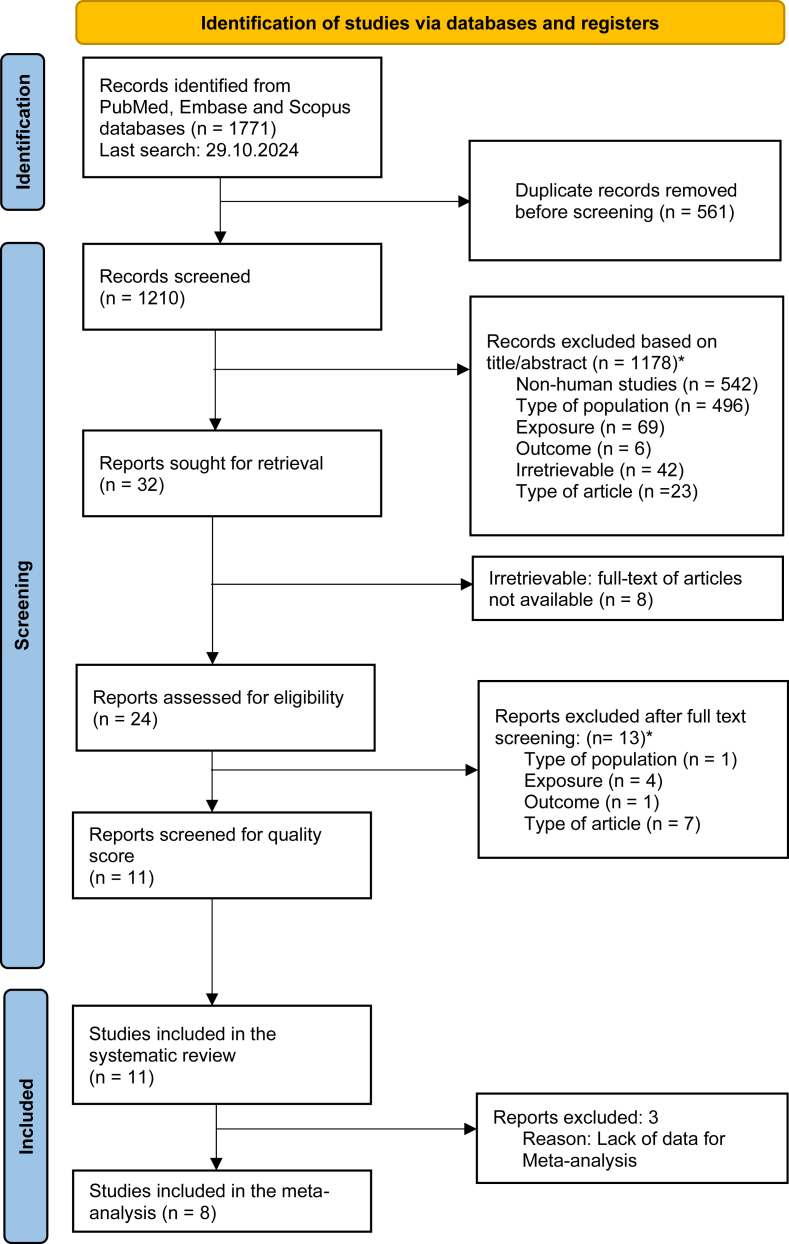
PRISMA flowchart of search and selection process. Exclusion criteria: non-human studies: animal studies or in vitro studies. population: age <18, females only cohort, azoospermic males, diseases such as cancer, among others. Exposure: Exposure is not Med Diet described using a priori indices. Outcome: does not have the outcomes of interest to the review (like semen parameters, fertilization outcomes, live birth, among others). Irretrievable: paper cannot be accessed. Type of article: reviews, opinion articles, case reports, letters, among others.

The PRISMA flowchart diagram is shown in Figure 1 depicts the process of article identification and exclusion. A total of 1771 articles were identified from PubMed, Embase, and Scopus databases, of which 561 were duplicates and removed. The remaining 1210 articles were screened by the title and abstract of the paper, and 1178 articles were excluded based on the inclusion and exclusion criteria mentioned before. Therefore, 32 papers were eligible for full-text screening of which only 24 could be retrieved. The other 8 articles did not have full text or the abstract. After excluding 13 articles that did not meet the inclusion criteria, only 11 articles were available for quality assessment. After analyzing the quality, 11 articles were finally included in the systematic review and 8 studies for meta-analysis, after excluding 3 articles because of lacking data after contacting with the corresponding authors without answer to our queries.

### Study characteristics

The characteristics of cross-sectional studies are described in Table 1 [8,18,19,21,[26], [27], [28], [29], [30], [31], [32], [33], [34], [35], [36], [37], [38], [39], [40], [41], [42], [43], [44], [45], [46], [47], [48], [49], [50], [51], [52], [53], [54], [55], [56], [57], [58], [59], [60], [61]] and those of RCTs and prospective cohort are presented in Table 2 [5,18,19,32,46,47,50,[52], [53], [54], [55], [56], [57]]. Of the 11 included studies, 8 were cross-sectional studies, 2 RCTs, and 1 was a prospective cohort study. All the studies included participants from the age range of 18–60 y. The studies were based in different countries, such as Italy (*n* = 4), Spain (*n* = 3), Greece (*n* = 1), Israel (*n* = 1), United States (*n* = 1), and Iraq (*n* = 1). The number of participants ranged from 50 to ∼300, and the studies included healthy males, infertile males, or males attending fertility clinics. In study quality assessments, 8 studies received a score of Fair (4–9), and 3 of them received a good score (>9) (TABLE 1, TABLE 2). A total of 2558 individuals from different studies were included in this SRMA.

**TABLE 1:** Primary outcomes related to adherence to Mediterranean diet.

	Reference	Sample size and population description	Country	Age, y	Type of study	Exposure	Diet pattern	Outcome	Main result	Quality assessment score
1.	Karayiannis et al. [26]	225 males from Couples undergoing fertility treatments	Greece	26–55	Cross-sectional study	The MedDiet adherence score was calculated with PMD [19], using the 75-item FFQ [36]	PMD	Volume, sperm concentration, total sperm count, total motility, progressive motility, morphology	A positive correlation of PMD score with semen quality parameters, where males in the highest tertile have significantly better sperm count, sperm concentration, total and progressive motility, and sperm morphology as compared with lower tertile males	9/14
2.	Efrat et al. [27]	280 males undergoing fertility treatments	Israel	18–55	Cross-sectional study	The MedDiet adherence score was calculated with HEI, AHEI, aMED, DASH [[37], [38], [39]] using 111-item FFQ [40,41]	HEI, AHEI, aMED, DASH	Semen parameters, including semen volume, sperm concentration, motility, total sperm count, and morphology	A positive correlation of aMED score with semen motility, where males in highest quartile had significantly higher motility, by 6% compared with males in the lowest quartile. There was no significant change reported in the sperm concentration, total sperm count, and morphology	8/14
3.	Salas-Huetos et al. [28]	106 young and healthy males	Spain	18–35	Cross-sectional study	The MedDiet adherence score was calculated with TMD [18] using 143-item FFQ [42]	TMD	pH, volume, total sperm count, concentration, vitality, total motility, progressive motility, nonprogressive motility, and morphology	A positive association of TMD score with sperm motility and semen pH, where males in the higher tertile had statistically significant higher total motility, higher progressive motility, and lower sperm immotility as compared with males in lower tertiles. There were no significant differences in the rest of the parameters	9/14
4.	Ricci et al. [29]	309 males undergoing fertility treatments	Italy	27–60	Cross-sectional study	The MedDiet adherence score was calculated with TMD score [18,43] using 78-item FFQ [44,45]	TMD, (Mediterranean Diet Score, MDS), developed by Trichopoulou et al. [18], modified for Italian dietary habits [43].	Semen volume, sperm concentration, and total sperm count	A positive association of TMD score with sperm concentration and count, where males in the highest tertile had statistically significant normal concentration and count as compared to those in the lowest category. There was no significant association with semen volume	8/14
5.	Cutillas-Tolín et al. [30]	209 young and healthy males	Spain	18–23	Cross-sectional study	The MedDiet adherence score was calculated with AHEI-2010 [46], rMED [21], and DASH [47] using 101-item FFQ [48,49]	AHEI-2010, rMED and DASH., (The rMED is a variation of the original MDS [18] created by Buckland et al. [21].	Sperm concentration, total sperm count, total motile sperm count, morphology, male reproductive hormones	A positive association between rMED score and FSH levels were observed. No association has been reported between rMED adherence and any of the semen parameters measured and other hormones tested (LH, estradiol, free testosterone, total testosterone, Inhibin B, and SHBG)	9/14
6.	Caruso et al. [31]	160 young and healthy males	Italy	18–30	RCT	—	—	Primary outcomes measured were changes in semen volume, sperm count, progressive motility, morphology, concentration, pH. Secondary outcomes were changes in weight (including BMI and waist circumference), glucose levels, blood lipids, and blood pressure	In the MedDiet intervention group, sperm concentration and sperm count both significantly increased after 6 mo as compared with baseline, but a significant increase was not reported in the low-fat diet group. The progressive motility increased in both groups individually but the differences within both groups were not significant. The decrease in sperm pH after 6 mo was significant in the low-fat diet group but not the MedDiet group. Semen volume and sperm morphology showed no change in either of the groups	10/14
7.	Salas-Huetos et al. [32]	245 males and their female partners undergoing fertility treatments	USA	18–55	Prospective cohort study	The MedDiet adherence score was calculated with the TMD [18], AMD [50], PMD [19], HEI [51], AHEI [46,52], AHA [53,54], DASH [47], and PBD [55] using the 131-item, FFQ [56,57]	TMD, AMD, PMD, HEI, AHEI, AHA, DASH, PBD	The primary outcome was live birth per treatment cycle. Secondary outcomes were fertilization, implantation, and clinical pregnancy and seminogram parameters	There was no association of several a priori-defined scores like TMD, PMD, and AMD, with MAR or semen quality	10/14
8.	Montano et al. [33]	263 young and healthy males	Italy	18–22	FASt Randomized Controlled Trial.	The MedDiet adherence score was calculated with [PREvención con DIeta MEDiterránea questionnaire (PREDIMED)] [58] and physical activity [International Physical Activity Questionnaire (IPAQ)] [59]	MEDAS	Primary outcomes were volume, sperm concentration, motility and morphology, concentration of round cells, and semen total antioxidant capacity	In the MedDiet intervention group, total and progressive motility, sperm concentration, and proportion of normal morphology cells increased but a decrease was observed in the control group, with statistically significant differences between the 2 groups at highest tertile after 4 mo of intervention	9/14
9.	Petre et al. [34]	300 males undergoing fertility treatments	Italy	18–45	Cross-Sectional Study	The MedDiet adherence score was calculated with a 14-point MEDAS questionnaire [60]	MEDAS	Semen volume, sperm concentration, total sperm count, sperm progressive motility, sperm viability, sperm typical morphology, semen pH, nonmotile sperm	A positive association of MEDAS with semen quality parameters, where males with medium and high MEDAS value had statistically significant higher total count, sperm concentration, viability, progressive motility, typical sperm morphology, and semen volume. There was no association with MEDAS and the semen pH and inverse association was found with nonmotile sperm	9/14
10.	Palani et al. [35]	261 males with normal and abnormal semen parameters	Iraq	22–56	Cross-sectional study	The MedDiet adherence score was calculated with aMED score [50] and using 147-item FFQ [61]	aMED	Semen volume, sperm concentration, sperm motility, progressive and nonprogressive, sperm number, sperm normal form, and viscosity	A positive association of aMED with semen quality parameters, where males in the highest tertile had a statistically significant increase in sperm concentration, total sperm number, total motility as compared with males in lower tertiles. There was no significant change in semen volume	8/14
11.	Davila-Cordova et al. [8]	200 young and healthy males	Spain	18–40	Cross-sectional study	The MedDiet adherence score was calculated with a validated 14-point MEDAS questionnaire [22] using a 143-item FFQ [42]	MEDAS	Sperm volume, sperm count and concentration, sperm vitality, total and progressive sperm motility, and sperm morphology	A positive association of MEDAS with semen quality parameters, where males in the highest tertile had a statistically significant higher sperm concentration, total sperm count, total motility, and progressive motility as compared with individuals in the lowest tertile. There was no association with the sperm morphology	10/14

Abbreviations: AHA, American Heart Association; AHEI, Alternative Healthy Eating Index; AMD, Alternate Mediterranean diet; aMED, alternate Mediterranean Diet score; DASH, Dietary Approaches to Stop Hypertension; FFQ, food frequency questionnaire; HEI, Healthy Eating Index; MEDAS, Mediterranean Diet Adherence Screener; MedDiet, Mediterranean diet; PBD, plant-based diet score; PMD, Panagiotakos Mediterranean diet; RCT, randomized clinical trial; rMED, relative Mediterranean diet score; SHBG, Sex Hormone Binding Globulin; TMD, Trichopoulou Mediterranean diet.

**TABLE 2:** Pregnancy and other fertility outcomes related to Mediterranean diet.

	Reference	Sample size and population description	Country	Age	Type of study	Exposure	Diet pattern	Outcome	Main result	Quality assessment score
**1.**	Salas-Huetos et al. [32]	245 males and their female partners undergoing fertility treatments	USA	18–55	Prospective cohort study	The MedDiet adherence score was calculated with the TMD [18], AMD [50], PMD [19], HEI [51], AHEI [46,52], AHA [53,54], DASH [47], and PBD [55] using the 131-item, FFQ [56,57]	TMD, AMD, PMD, HEI, AHEI, AHA, DASH, PBD	The primary outcome was live birth per treatment cycle. Secondary outcomes were fertilization, implantation, and clinical pregnancy and seminogram parameters	There was no association of several a priori-defined scores like TMD, PMD, and AMD, with MAR or semen quality	10/14

Abbreviations: AHA, American Heart Association; AHEI, Alternative Healthy Eating Index; AMD, Alternate Mediterranean diet; aMED, alternate Mediterranean Diet score; DASH, Dietary Approaches to Stop Hypertension; FFQ, food frequency questionnaire; HEI, Healthy Eating Index; MEDAS, Mediterranean Diet Adherence Screener; MedDiet, Mediterranean diet; PBD, plant-based diet score; PMD, Panagiotakos Mediterranean diet; RCT, randomized clinical trial; rMED, relative Mediterranean diet score; TMD, Trichopoulou Mediterranean diet.

### Qualitative synthesis

#### Cross-sectional studies

All 8 cross-sectional studies checked the association between adherence to the MedDiet and sperm concentration and count. A total of 5 studies reported a positive association between MedDiet adherence and sperm concentration and count. Three of them (Karayiannis et al. [26] using the PMD score; Petre et al. [34] using the MEDAS questionnaire; Ricci et al. [29] using the TMD score) were conducted in individuals recruited in fertility clinics, and in 2 studies (Davila-Cordova et al. [8] using the MEDAS questionnaire; Palani et al. [35] using the aMED score) participants were recruited from healthy well-being population. No associations between MedDiet adherence and these sperm variables were reported in other 3 studies—2 conducted in healthy individuals (Cutillas-Tolín et al. [30] using the rMED score; Salas-Huetos et al. [28] using the TMD score) and 1 in individuals recruited from fertility clinics (Efrat et al. [27] using the aMED score).

Seven articles have assessed associations between adherence to the MedDiet and sperm motility-related parameters (including total progressive motility or immotility). A total of 5 or 4 studies reported a positive association of MedDiet adherence with total motility [8,[26], [27], [28],^35^] or progressive motility [8,26,28,34], respectively. Three of these studies were conducted on males from fertility clinics [26,27,34] and 3 in a young healthy population [8,28,35]. An inverse association between MedDiet adherence and sperm immotility was reported in studies performed in young healthy individuals [28] or fertility clinic participants [34]. No significant associations with motility were reported by Cutillas-Tolín et al. [30] in young healthy participants.

The association between MedDiet adherence and sperm morphology (or sperm normal form) was determined in 6 studies. In 2 of them (in both studies volunteers from fertility clinics), a positive association with normal sperm morphology was reported [26,34] and other 4 reported no significant association for the morphology parameter—1 study conducted in fertility clinics [27] and the other 3 in healthy participants- [8,28,30].

The association between MedDiet adherence and semen volume was analyzed by 3 studies. One study conducted on males from the fertility clinic, reported a significant positive association [34] whereas the other 2 studies reported no association: one study recruited males from fertility clinics [29] and the other enrolled individuals from the healthy population [35].

Only 2 studies that recruited males from a young healthy population have analyzed the association of adherence to MedDiet with sperm vitality, being the association nonsignificant in any of the studies [8,28]. Another study analyzed vitality in individuals from a fertility clinic, showing a significant positive association with the degree of adherence to MedDiet [34].

Male reproductive hormones were measured in individuals from a young and healthy population as one of the outcomes by Cutillas-Tolín et al. [30], there was a positive association of FSH concentrations with MedDiet adherence, and no association was reported for other hormones measured.

Concerning semen pH, only 1 study conducted on males from a fertility clinic showed no association with MedDiet adherence [34].

In summary, the results of these cross-sectional studies suggest that adherence to MedDiet significantly influences increased sperm motility, progressive motility, concentration, and count. Other seminal parameters might need further research and evidence to establish clear associations.

#### Prospective studies

Our review included 1 prospective cohort study by Salas-Huetos et al. [32] conducted on 245 males and their female partners who underwent MAR cycles. The authors analyzed the associations between 3 MedDiet scores (TMD, AMD, and PMD) and different major outcomes (live birth per assisted reproductive treatment cycle and fertilization, implantation, clinical pregnancy, and seminogram parameters). None of the MedDiet scores were associated with the MAR success, or with the semen quality parameters analyzed (ejaculate volume, sperm count, concentration, total motility, progressive motility, and normal sperm morphology).

#### RCTs

Two RCTs conducted on healthy young males were reported in the literature suggesting that MedDiet ameliorate some semen quality parameters.

In the 1st one, conducted by Montano et al. [33], the effect of a lifestyle intervention promoting a MedDiet and physical activity or a control intervention (receiving written national dietary guidelines) on several sperm parameters was assessed in 263 healthy young males from 3 areas of Italy, using a parallel design RCT. After 16 weeks, an increase in sperm concentration, total and progressive motility, and proportion of normal morphology sperm cells was shown in the intervention group, whereas these parameters decreased in the control group. Significant differences in changes between groups were also reported for adherence to MedDiet, total motility, progressive motility, and semen total antioxidant capacity.

In 2 RCTs by Caruso et al. [28], in young healthy males from Italy (*n* = 160), the effect on the seminal parameters was assessed between participants following a MedDiet or a low-fat diet. After 6 mo of intervention, the sperm concentration and sperm count increased only in the MedDiet group, with the differences in changes between groups being statistically significant. As for progressive motility, the increase was significant in participants from both intervention groups, but no significant between group differences in changes were reported. Participants from both intervention groups showed a 6-mo decrease in semen pH, without significant between intervention effects. Semen volume decreased after the low-fat diet, but no differences in the effect between groups were shown.

### Quantitative analysis

**FIGURE 2:**
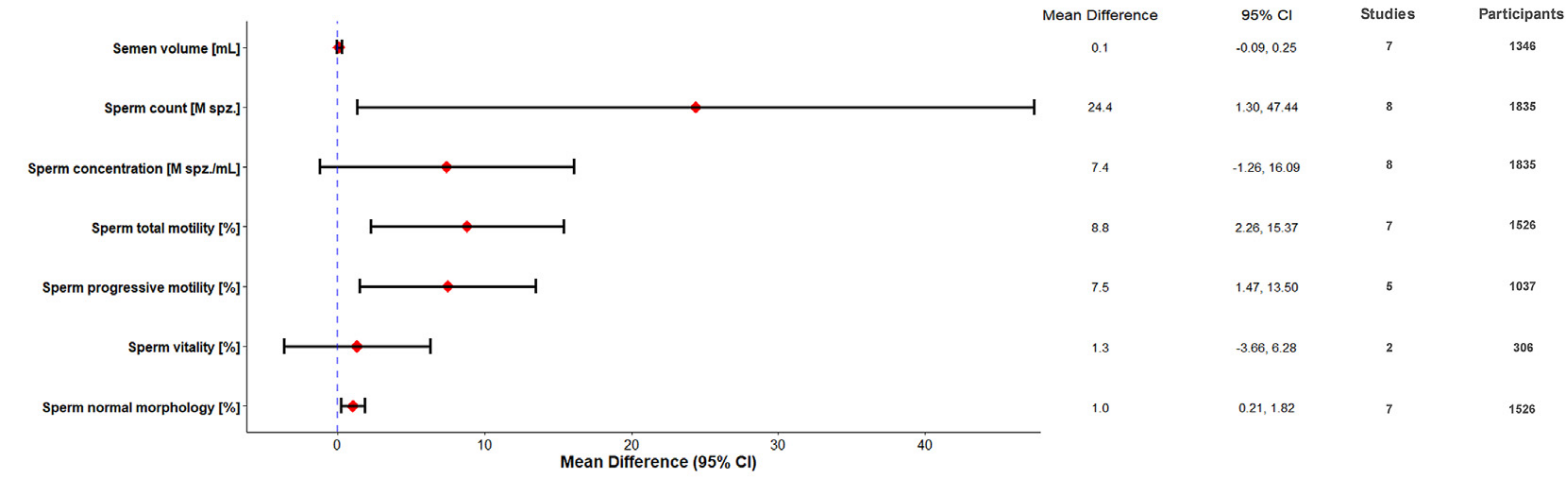
Forest superplot of pooled mean differences (MD) for studies showing the association between total MedDiet and semen quality parameters. CI confidence interval; MedDiet, Mediterranean diet; M spz., million spermatozoa; M spz., spermatozoa.

In Figure 2, we show the super plot summarizing the total MedDiet meta-analysis conducted for each of the semen parameters using cross-sectional and prospective cohort studies. A total of 1835 individuals from 8 observational studies were included in the meta-analysis. The results of the 2 RCTs were not meta-analyzed because of the intervention time difference between the articles including different spermatogenesis cycles.

#### Total MedDiet and sperm parameters

The results for combined total MedDiet adherence studies (Supplemental Figure 1 and Figure 2) revealed significant positive association for sperm count (24.37 M spz.; 1.30–47.44 M spz.; *P* = 0.04), total motility (8.81%; 2.26%–15.37%; *P* = 0.008), progressive motility (7.49%; 1.47–13.50 %; *P* = 0.01), and sperm normal morphology (1.02%,; 0.21%–1.82%;, *P* = 0.01), suggesting that higher MedDiet adherence is beneficial for some semen quality parameters. In contrast, nonsignificant positive associations were shown for semen volume (0.08 mL; –0.09–0.25 mL; *P* = 0.38), sperm concentration (7.41 M spz./mL; −1.26 to 16.09 M spz./mL; *P* = 0.09), and sperm vitality (1.31%; −3.66 to 6.28%; *P* = 0.61). The heterogeneity among studies was deemed substantial for semen volume (*I*^2^ = 53%; *P* = 0.04) and for sperm vitality (*I*^2^ = 50%; *P* = 0.16), and considerable for sperm count (*I*^2^ = 89%; *P* < 0.00001), sperm concentration (*I*^2^ = 89%; *P* < 0.00001), sperm total motility (*I*^2^ = 88%; *P* < 0.00001), sperm progressive motility (*I*^2^ = 86%; *P* < 0.00001), and sperm normal morphology (*I*^2^ = 77%; *P* < 0.0001).

Systematic exclusion of 1 article at a time for each semen quality parameter was checked showing the changes for MD and *I*^2^ (Supplemental Table 4). The values for the association of total MedDiet with semen volume explained heterogeneity changes from substantial to moderate after removing data from Salas-Huetos et al. [32], total Mediterranean diet and sperm parameters using the PMD or TMD score. Association between total MedDiet and sperm count became nonsignificant after the removal of data from Karayiannis et al. [26] (PMD), Cutillas-Tolín et al. [30] (rMED), Salas-Huetos et al. [28] (TMD), Palani et al. [35] (AMD), or Davila-Cordova et al. [8] (MEDAS), heterogeneity was changed from considerable to substantial by excluding values from Palani et al. [35] (AMD). For sperm concentration, heterogeneity was changed from considerable to moderate, and MD was reduced by >20% when the Palani et al.’s [35] (AMD) results were excluded from the analysis. The association between total MedDiet and sperm total motility explained heterogeneity change from considerable to substantial by removing data from Palani et al. [35] (AMD). Similarly, for sperm progressive motility, the change in heterogeneity from considerable to moderate was explained, and MD was reduced by >20% by removing values from Palani et al. [35] (AMD). After removing the Palani et al. [35] (AMD) study, the heterogeneity in the association between total MedDiet and sperm normal morphology changed from considerable to homogenous with *I*^2^ = 0 and >20% reduction in MD. Overall, the study by Palani et al. [35] (AMD) was highly influential. The funnel plot also shows symmetry except for Palani et al. [35], which has heterogeneous data and stands out as an outlier (Supplemental Figure 2).

A statistically significant positive association between total MedDiet adherence and semen volume, sperm count, sperm concentration, total or progressive motility, and morphology was shown (not in case of vitality) for those individuals recruited from healthy population (Supplemental Figure 3). The funnel plot for healthy participants visually indicates that the study by Palani et al. [35] is contributing to the asymmetry and is the most heterogeneous study (Supplemental Figure 4). Positive associations with sperm concentration, total motility, and morphology were also observed for those participants recruited from fertility clinics, but not for the other semen quality parameters analyzed (Supplemental Figure 5). The funnel plot representation for participants from fertility clinics was mostly symmetrical (Supplemental Figure 6).

#### TMD and sperm parameters

TMD diet score was positively associated to semen volume (0.15 mL; 0.07–0.23 mL; *P* = 0.0001), sperm count (17.08 M spz.; 11.38–22.7 M spz.; *P* < 0.00001), sperm concentration (6.57 M spz./mL; 4.47–8.67 M spz./mL; *P* < 0.00001), sperm total motility (9.01%; 2.75%–15.26%; *P* = 0.005), and sperm normal morphology (0.74%; 0.04%–1.45%; *P* = 0.04). Nonsignificant positive association for sperm progressive motility (3.84%; −0.91 to 8.58%; *P* = 0.11), was also observed. The heterogeneity among studies was deemed to be moderate for sperm concentration (*I*^2^ = 45%; *P* = 0.16) and sperm normal morphology (*I*^2^ = 14%; *P* = 0.28) and substantial for semen volume (*I*^2^ = 63%,; *P* = 0.07), sperm count (*I*^2^ = 67%, *P* = 0.05), and sperm progressive motility (*I*^2^ = 63%; *P* = 0.10). In case of total motility, the heterogeneity was considerable (*I*^2^ = 80%; *P* = 0.02) (Supplemental Figure 7).

#### AMD index and sperm parameters

The results of studies analyzing the AMD diet score showed a positive association for sperm count (32.28 M spz.; 15.86–48.69 M spz.; *P* = 0.0001), sperm concentration (18.77 M spz./mL; 13.02–24.52 M spz./mL; *P* < 0.00001), sperm total motility (10.93%; 6.97%–14.89%; *P* < 0.00001), sperm progressive motility (8.83%; 4.87−12.79; *P* < 0.0001), and sperm normal morphology (2.16%; 1.61%–2.71%; *P* < 0.00001). Nonsignificant positive association for semen volume (0.14 mL; −0.14 to 0.42 mL; *P* = 0.33) was shown. The heterogeneity among studies was substantial for semen volume (*I*^2^ = 60%; *P* = 0.11) and considerable for sperm count (*I*^2^ = 97%; *P* < 0.00001), sperm concentration (*I*^2^ = 97%; *P* < 0.00001), sperm total motility (*I*^2^ = 95%; *P* < 0.00001), sperm progressive motility (*I*^2^ = 97%; *P* < 0.00001), and sperm normal morphology (*I*^2^ = 87%, *P* < 0.00001) (Supplemental Figure 8).

#### PMD and sperm parameters

Positive association between adherence to MedDiet using the PMD score and sperm normal morphology (0.70%; 0.06%–1.34%; *P* = 0.03) and sperm total motility (5.94%; 0.75%–11.13%; *P* = 0.02) was shown. In contrast, no significant associations were observed for semen volume (–0.06 mL; −0.31% to 0.19%; *P* = 0.66), sperm count (5.48 M spz.; −13.40 to 24.36 M spz.; *P* = 0.57), sperm concentration (5.09 M spz./mL; −2.03 to 12.48 M spz./mL; *P* = 0.18), and sperm progressive motility (2.45%; −1.14% to 6.04%; *P* = 0.18). The heterogeneity among studies was deemed to be substantial for semen volume (*I*^2^ = 57%; *P* = 0.13), sperm count (*I*^2^ = 57%; *P* = 0.13), sperm progressive motility (*I*^2^ = 64%; *P* = 0.10); and for sperm normal morphology (*I*^2^ = 58%; *P* = 0.12), considerable for sperm total motility (*I*^2^ = 75%; *P* = 0.04), and homogenous for sperm concentration (*I*^2^ = 0%; *P* = 0.37) (Supplemental Figure 9).

## Discussion

This SRMA including 11 studies provides expanded and updated data concerning the relationship between adherence to MedDiet and semen quality parameters, as well as other male fertility indicators, being the most updated and comprehensive meta-analysis on this topic. Specifically, in this meta-analysis of the 8 observational studies, MedDiet adherence showed a significant positive association with sperm count, total motility, progressive motility, and sperm normal morphology. The results were consistent with the primary analysis when the exposure was tested using the different MedDiet scores and checking the influence of each of the studies on these associations. Only 1 study reported a positive association between MedDiet adherence and FSH concentrations. The association between MedDiet adherence using different scores and MAR success or semen quality outcomes was only studied in 1 study, showing no associations. Evidence from the 2 RCTs analyzing the effect of MedDiet on semen parameters align with the results of the meta-analysis conducted using data from observational studies as they have demonstrated beneficial effects of MedDiet on total and progressive motility in one of the studies and sperm concentration and count in the other one.

In our study, we have also conducted subgroup meta-analyses in relation to the type of individuals included. It is important to highlight that the positive associations between total MedDiet adherence and sperm concentration, total motility, and morphology were observed for healthy but also for those participants recruited in fertility clinics, suggesting that MedDiet may have a positive impact on semen parameters in healthy but also in specific populations including individuals with fertility difficulties. The studies with healthy participants had more semen quality parameters positively associated with adherence to MedDiet than those with fertility clinic participants. This can be explained because, compared with healthy participants, males recruited in fertility clinics may have more probabilities to be infertile because of psychological stress [62], or other causes beyond of lifestyle or other modifiable risk factors of infertility, such as genetic causes [63,64], infection diseases [65], among others.

Three previous reviews and meta-analyses on this topic have been conducted, showing some limitations or differences in criteria for inclusion of studies as compared with our present review. Cao et al. [13] meta-analyzed 6 cross-sectional studies testing the association between different dietary patterns (MedDiet, DASH, and Prudent diet) and semen parameters without including analysis of MAR outcomes and results from clinical trials. They reported that a healthy diet, including the MedDiet, in general was positively associated with sperm concentration, progressive sperm motility, and total sperm count. The SRMA of Muffone et al. [14] focused on female-related outcomes, and meta-analyzed 3 studies focusing in male outcomes showing an association between MedDiet and live birth, pregnancy rate, sperm concentration, and sperm count, but no consistent results in meta-analysis were found. The 3rd study by Piera-Jordan et al. [12,15] was a systematic review without meta-analysis focusing on dietary patterns, nutrients, and food groups, including only 5 cross-sectional, 1 prospective cohort observational studies, and 1 clinical trial analyzing the association between MedDiet adherence and semen quality parameters. Therefore, the present SRMA is unique, as it was focused on the relationship between male MedDiet adherence, semen quality parameters, and other fertility outcomes, including 9 observational studies (some of them recently published) and 2 clinical trials. In addition, our SRMA also provide for the first-time information in relation to the type of individuals (healthy compared with from fertility clinics).

The MedDiet provides numerous benefits, including reducing the risk of various chronic diseases [66] and increasing life expectancy [67], with each nutrient or food playing a unique role through specific metabolic pathways. This dietary pattern is rich in various nutrients like unsaturated fats [monounsaturated fatty acids (MUFAs) and omega-3 fatty acids (ω-3 fatty acids)], antioxidants (polyphenols, vitamin E, carotenoids, vitamin C, selenium, zinc, among others), folate, minerals (for example, magnesium, calcium, and potassium), dietary fiber, and has a low glycemic index [68,69]. The mechanisms underlying the beneficial effects of the MedDiet on semen quality and fertility are likely multiple and synergistic. Some important ones have been described recently using a nonvalidated definition of MedDiet (for example, increasing testosterone concentrations or reducing DNA fragmentation indices) [70]. Unsaturated fatty acids, like ω-3 fatty acids—which are a type of polyunsaturated fatty acids—are incorporated into the spermatozoa membrane, where they play a vital role in supporting fertilization [71]. A proinflammatory and oxidative status has been recognized as a potential mechanism disrupting spermatogenesis [72], and MUFAs are less prone to be oxidized. Several antioxidants like polyphenols are essential for fertility as they prevent sperm DNA damage by scavenging reactive oxygen species [73], selenium removes hydrogen peroxide by increasing the activity of glutathione peroxidase-1 [74], and zinc seams essential for stabilizing sperm membrane, and controlling sperm DNA condensation and decondensation [75]. Folate, a naturally occurring form of Vitamin B9, found mainly in green leafy vegetables and fruits, plays an essential role in spermatogenesis by reducing the incidence of sperm aneuploidy [76]. Few studies have examined the effects of dietary fiber on male infertility. However, it has been suggested that fiber binds to unconjugated estrogen [77], and maintaining low plasma estrogen concentrations is essential for normal male fertility [78]. Also, diets low on glycemic index help lower insulin resistance risk and stabilize blood sugar concentrations. Some studies have shown that the glycemic index was negatively associated with changes in total sperm count and total motility [77] and decreased fecundability [79]. Collectively the above findings suggest that MedDiet could be a holistic approach to improve fertility outcomes by balancing peripheral reproductive hormones, reducing oxidative stress, and consequently minimizing sperm DNA damage, and enhancing overall metabolic health.

This SRMA has several strengths. First, it includes more studies than the previous SRMAs already conducted as represent the last update on this subject. Although prior reviews have explored this topic, they have often been limited by narrow populations (such as only fertility clinic patients or only healthy participants), outdated data (despite the field’s rapid evolution and the high relevance of recent publications), or methodological shortcomings (such as incorrectly combining a priori and a posteriori dietary patterns results). This review addresses these limitations by incorporating the most up-to-date studies and systematically synthesizing findings across diverse populations and approaches, uncovering patterns, gaps, and contradictions that have not been previously explored. Second, it is focused on analyzing specific association of a priori MedDiet measured adherence with seminal quality parameters, peripheral hormone concentrations, and MAR outcomes, including observational studies but also RCTs. Third, we assessed the quality of the included studies using the NHLBI tool, which is particularly well-suited for observational studies, the predominant study type in our review, making it the most appropriate choice for this assessment. However, our SRMA had also some limitations that should be acknowledged. We were not able to include 1 of the already published observational study in the meta-analysis because of a lack of data provided, and unfortunately, we cannot meta-analyze the results of the only 2 published clinical trials results because of the heterogeneity between them. Another important limitation is the intrinsic use food frequency questionnaires and dietary indexes of the papers included because all the results rely on self-reported data, making them prone to recall bias and misreporting, which can compromise the accuracy of dietary assessment. It is also important to note that semen quality measurements should be considered as a subrogated indicator of fertility status, this is because they often do not strongly correlate with key fertility outcomes, such as clinical pregnancy or live birth rates. Furthermore, because most of the studies systematically reviewed were observational, causality cannot be inferred and we cannot rule out the possibility that other lifestyle factors may also influence the results.

In conclusion, our study gives a specific and updated overview of the associations between adherence to MedDiet, semen quality, peripheral hormone concentrations, and MAR outcomes in the male population. The results suggest that MedDiet has protective effects on semen quality parameters on both participants from healthy populations and fertility clinics, although more significant associations were obtained evaluating healthy populations. However, no robust evidence exists in relation to potential beneficial effects of MedDiet on MAR outcomes. Future research is needed to strength the scientific evidence and clinical relevance in relation of the effect of MedDiet on fertility using RCT with appropriate large samples and follow-up measuring not only sperm parameters as a surrogate endpoint of fertility. In addition, feasibility trials conducted in other non-Mediterranean populations are needed to demonstrate that changes in the dietary pattern are possible at long term in other populations. Despite more studies are required, including nutrition experts within the multidisciplinary team of fertility professionals to assess dietary habits might help improve semen parameters.

## Author contributions

The authors’ responsibilities were as follows – RA, JS-S, AS-H: designed research; RA, JS-S, ED-C, SS, AS-H: conducted research; RA, ED-C, SS, MPA, AS-H: analyzed data or performed statistical analysis; RA: wrote and revised the paper; and all authors: had primary responsibility for final content, reviewed the results, and approved the final version of the manuscript.

## Data availability

The datasets generated during and/or analyzed during this study are available from the corresponding author on reasonable request.

## Funding

Supported by Instituto de Salud Carlos III through the project PI21/01447 (co-funded by the European Union) and partially supported by Diputació de Tarragona (2021/11–No.Exp.8004330008–2021–0022642). This work was partially supported by ICREA under the ICREA Academia program (JS-S). RA was supported by a predoctoral grant from the Agència de Gestió d'Ajuts Universitaris i de Recerca (AGAUR) and Generalitat de Catalunya. SS is supported by a Miguel Servet Contract from the Instituto de Salud Carlos III (CP24-0006), Spain and cofinanced by the European Union. ED-C has received a Contrato Pre-doctoral de Formación en Investigación en Salud (PFIS FI22/00018) of the Acción Estratégica en Salud program (AES) from the Carlos III Health Institute (ISCIII), Spanish Ministry of Health.

## Conflict of interest

The authors declare no conflicts of interest.
