# Supplementary material for: Mediterranean Diet, Semen Quality, and Medically Assisted Reproductive Outcomes in the Male Population: A Systematic Review and Meta-Analysis
Source: Adv Nutr. 2025 May 24;16(8):100454. doi: 10.1016/j.advnut.2025.100454 (PMC12276387; doi:10.1016/j.advnut.2025.100454)
Supplement: Multimedia component 1 [file mmc1.docx]

# **Supplemental material**

## Mediterranean diet, semen quality and medically assisted reproductive outcomes in the male population: a systematic review and meta-analysis

Rashmi Agarwal

# **List of Tables and Figures**

**Supplemental Table 1.** Keywords used for literature search in the PUBMED, EMABSE and SCOPUS databases

**Supplemental Table 2.** PECOS (Population, Exposure, Comparison, Outcomes, Study design, Article type) for the inclusion and exclusion criteria. (MAR: Medically assisted reproductive)

**Supplemental Table 3.** Mediterranean dietary pattern components and scoring methodologies.

**Supplemental Table 4.** Sensitivity analysis by systematic exclusion of one study at a time (sorted by author).

**Supplemental Figure 1.** Forest plot of mean differences (MD) and 95% confidence intervals (CI) for studies evaluating the association between adherence to Total MedDiet and (A) Semen volume [mL], (B) Sperm count [M spz.], (C) Sperm concentration [M spz./mL], (D) Sperm total motility [%], (E) Sperm progressive motility [%], (F) Sperm vitality [%], (G) Sperm normal morphology [%].

**Supplemental Figure 2.** Funnel plot of the meta-analysis plotted with standard error (SE) on the y-axis and mean difference (MD) on the x-axis, evaluating the association between adherence to Total MedDiet and (A) Semen volume [mL], (B) Sperm count [M spz.], (C) Sperm concentration [M spz./mL], (D) Sperm total motility [%], (E) Sperm progressive motility [%], (F) Sperm vitality [%], (G) Sperm normal morphology [%].

**Supplemental Figure 3.** Forest plot of mean differences (MD) and 95% confidence intervals (CI) for studies evaluating the association between adherence to Total MedDiet and (A) Semen volume [mL], (B) Sperm count [M spz.], (C) Sperm concentration [M spz./mL], (D) Sperm total motility [%], (E) Sperm progressive motility [%], (F) Sperm vitality [%], (G) Sperm normal morphology [%] in the participants from Healthy population.

**Supplemental Figure 4.** Funnel plot of the meta-analysis plotted with standard error (SE) on the y-axis and mean difference (MD) on the x-axis, evaluating the association between adherence to Total MedDiet and (A) Semen volume [mL], (B) Sperm count [M spz.], (C) Sperm concentration [M spz./mL], (D) Sperm total motility [%], (E) Sperm progressive motility [%], (F) Sperm vitality [%], (G) Sperm normal morphology [%] in the participants from Healthy population.

**Supplemental Figure 5.** Forest plot of mean differences (MD) and 95% confidence intervals (CI) for studies evaluating the association between adherence to Total MedDiet pattern and (A) Semen volume [mL], (B) Sperm count [M spz.], (C) Sperm concentration [M spz./mL], (D) Sperm total motility [%], (E) Sperm progressive motility [%], (F) Sperm normal morphology [%] in participants recruited from fertility clinics.

**Supplemental Figure 6.** Funnel plot of the meta-analysis plotted with standard error (SE) on the y-axis and mean difference (MD) on the x-axis, evaluating the association between adherence to Total MedDiet pattern and (A) Semen volume [mL], (B) Sperm count [M spz.], (C) Sperm concentration [M spz./mL], (D) Sperm total motility [%], (E) Sperm progressive motility [%], (F) Sperm normal morphology [%] in participants recruited from fertility clinics.

**Supplemental Figure 7.** Forest plot of mean differences (MD) and 95% confidence intervals (CI) for studies evaluating the association between adherence to TMD diet pattern and (A) Semen volume [mL], (B) Sperm count [M spz.], (C) Sperm concentration [M spz./mL], (D) Sperm total motility [%], (E) Sperm progressive motility [%], (F) Sperm normal morphology [%].

**Supplemental Figure 8.** Forest plot of mean differences (MD) and 95% confidence intervals (CI) for studies evaluating the association between adherence to AMD diet pattern and (A) Semen volume [mL], (B) Sperm count [M spz.], (C) Sperm concentration [M spz./mL], (D) Sperm total motility [%], (E) Sperm progressive motility [%], (F) Sperm normal morphology [%].

**Supplemental Figure 9.** Forest plot of mean differences (MD) and 95% confidence intervals (CI) for studies evaluating the association between adherence to PMD diet pattern and (A) Semen volume [mL], (B) Sperm count [M spz.], (C) Sperm concentration [M spz./mL], (D) Sperm total motility [%], (E) Sperm progressive motility [%], (F) Sperm normal morphology [%].

**Supplemental Table 1.** Keywords used for literature search in the PUBMED, EMABSE and SCOPUS databases

| Searches in PubMed | ((((Mediterranean diet[Title/Abstract]) OR (mediterranean[Title/Abstract])) OR (Mediterranean diet[MeSH Terms])) OR (Mediterranean diet*[Title/Abstract]))  AND ((((((((((((((((((((((((((((((((((((((((((((((((((((((((((((((((Semen[Title/Abstract]) OR  (Sperm[Title/Abstract])) OR (semen quality[Title/Abstract])) OR (sperm quality[Title/Abstract])) OR (Semen Qualities[Title/Abstract])) OR (Semen Analysis[Title/Abstract])) OR (Semen Analyses[Title/Abstract])) OR (semen parameters[Title/Abstract])) OR (seminogram parameters[Title/Abstract])) OR (sperm parameters[Title/Abstract])) OR (semen volume[Title/Abstract])) OR (semen pH[Title/Abstract])) OR (sperm concentration[Title/Abstract])) OR (concentration of round cells[Title/Abstract])) OR (sperm count[Title/Abstract])) OR (total sperm number[Title/Abstract])) OR (sperm motility[Title/Abstract])) OR (total motility[Title/Abstract])) OR (progressive motility[Title/Abstract])) OR (non-progressive motility[Title/Abstract])) OR (total motile sperm count[Title/Abstract])) OR (non-motile sperm[Title/Abstract])) OR (sperm morphology[Title/Abstract])) OR (morphological abnormalities[Title/Abstract])) OR (sperm normal forms[Title/Abstract])) OR (sperm vitality[Title/Abstract])) OR (sperm viability[Title/Abstract])) OR (spermatozoa[Title/Abstract])) OR (sperm function[Title/Abstract])) OR (sperm tail[Title/Abstract])) OR (hormones in male reproductive[Title/Abstract])) OR (testosterone[Title/Abstract])) OR (Total testosterone[Title/Abstract])) OR (Free testosterone[Title/Abstract])) OR (Estradiol[Title/Abstract])) OR (Inhibin-B[Title/Abstract])) OR (LH[Title/Abstract])) OR (Luteinizing hormone[Title/Abstract])) OR (FSH[Title/Abstract])) OR (Follicle-Stimulating Hormone[Title/Abstract])) OR (SHBG[Title/Abstract])) OR (Sex hormone-binding globulin[Title/Abstract])) OR (live birth[Title/Abstract])) OR (pregnancy outcomes[Title/Abstract])) OR (fertilization[Title/Abstract])) OR (implantation[Title/Abstract])) OR (clinical pregnancy[Title/Abstract])) OR (pregnancy loss[Title/Abstract])) OR (miscarriage[Title/Abstract])) OR (assisted reproduction[Title/Abstract])) OR (Intracytoplasmic Sperm Injection[Title/Abstract])) OR (Fertilization in Vitro[Title/Abstract])) OR (IVF[Title/Abstract])) OR (in vitro fertilization[Title/Abstract])) OR (Reproductive Techniques[Title/Abstract])) OR (Medically Assisted Reproduction[Title/Abstract])) OR (Assisted Reproductive Technique[Title/Abstract])) OR (Assisted Reproductive Technology[Title/Abstract])) OR (male infertility[Title/Abstract])) OR (Male sterility[Title/Abstract])) OR (Male Subfertility[Title/Abstract])) OR (semen total antioxidant capacity[Title/Abstract])) OR (sperm DNA fragmentation[Title/Abstract])) OR (sperm DNA damage[Title/Abstract])) |
| --- | --- |
| Searches in EMBASE | ('mediterranean diet':ab,ti OR 'mediterranean':ab,ti)  AND  ('sperm':ab,ti OR 'sperm quality':ab,ti OR 'semen analysis':ab,ti OR 'semen parameters':ab,ti OR 'spermiogram':ab,ti OR 'semen volume':ab,ti OR 'semen ph':ab,ti OR 'sperm count':ab,ti OR 'progressive motility':ab,ti OR 'total motile sperm count':ab,ti OR 'sperm morphology':ab,ti OR 'sperm viability':ab,ti OR 'sperm function':ab,ti OR 'testosterone':ab,ti OR 'total testosterone':ab,ti OR 'estradiol':ab,ti OR 'inhibin b':ab,ti OR 'luteinizing hormone':ab,ti OR 'sex hormone binding globulin':ab,ti OR 'live birth':ti,ab OR 'pregnancy outcomes':ab,ti OR 'fertilization':ab,ti OR 'implantation':ab,ti OR 'clinical pregnancy':ab,ti OR 'assisted reproduction':ab,ti OR 'intracytoplasmic sperm injection':ab,ti OR 'in vitro fertilization':ab,ti OR 'ivf':ab,ti OR 'medically assisted reproduction':ab,ti OR 'male infertility':ab,ti OR 'male sterility':ab,ti OR 'male subfertility':ab,ti OR 'sperm dna fragmentation':ab,ti OR 'sperm dna damage':ab,ti) |
| Searches in Scopus | ( TITLE-ABS-KEY ( ( semen AND qualities ) OR ( semen AND analysis ) OR ( semen AND analyses ) OR ( semen AND parameters ) OR ( seminogram AND parameters ) OR ( sperm AND parameters ) OR ( semen AND volume ) OR ( semen AND ph ) OR ( sperm AND concentration ) OR ( concentration AND of AND round AND cells ) OR ( sperm AND count ) OR ( total AND sperm AND number ) OR ( sperm AND motility ) OR ( total AND motility ) OR ( progressive AND motility ) OR ( non-progressive AND motility ) OR ( total AND motile AND sperm AND count ) OR ( non-motile AND sperm ) OR ( sperm AND morphology ) OR ( morphological AND abnormalities ) OR ( sperm AND normal AND forms ) OR ( sperm AND vitality ) OR ( sperm AND viability ) OR ( spermatozoa OR sperm AND function ) OR ( sperm AND tail ) OR ( hormones AND male AND reproductive ) OR ( testosterone ) OR ( total AND testosterone ) OR ( free AND testosterone ) OR ( ( estradiol OR inhibin-b OR lh OR luteinizing ) AND hormone ) OR ( fsh OR follicle-stimulating AND hormone ) OR ( shbg OR sex AND hormone-binding ) AND ( globulin ) OR ( live AND birth ) OR ( pregnancy AND outcomes ) OR ( fertilization ) OR ( implantation ) OR ( clinical AND pregnancy ) OR ( pregnancy AND loss ) OR ( miscarriage ) OR ( assisted AND reproduction ) OR ( intracytoplasmic AND sperm AND injection ) OR ( fertilization AND in AND vitro ) OR ( ivf ) OR ( in AND vitro AND fertilization ) OR ( reproductive AND techniques ) OR ( medically AND assisted AND reproduction ) OR ( assisted AND reproductive AND technique ) OR ( assisted AND reproductive AND technology ) OR ( male AND infertility ) OR ( male AND sterility ) OR ( male AND subfertility ) OR ( semen AND total AND antioxidant AND capacity ) OR ( sperm AND dna AND fragmentation ) OR ( sperm AND dna AND damage ) ) AND TITLE-ABS-KEY ( mediterranean AND diet ) AND NOT TITLE ( review ) ) AND PUBYEAR > 2014 AND PUBYEAR < 2025 AND ( LIMIT-TO ( SRCTYPE , "j" ) ) AND ( LIMIT-TO ( DOCTYPE , "ar" ) ) AND ( LIMIT-TO ( LANGUAGE , "English" ) ) |

| **Parameter** | **Inclusion** | **Exclusion** |
| --- | --- | --- |
| **Population** | - Male of reproductive age - Humans | - Less than 18 years old - Women participants only - Azoospermic men - Medication (e.g., phosphodiesterase type 5 inhibitors) - Alcohol or drug abuse - Severe diseases (i.e., cancer, chronic illness) |
| **Exposure** | - Mediterranean Diet (*a priori*): - Trichopoulus Mediterranean Diet Score - Panagiotakos Mediterranean Diet Score - Relative Mediterranean Diet Score - Alternate Mediterranean Diet Score | - *A posteriori* studies - Other dietary patterns beyond the Mediterranean Diet |
| **Comparison** | - Low adherence vs. High adherence | - Studies with low sample size (n<20 per group) - Studies with poorly defined methodology |
| **Outcomes** | - Primary outcomes: SeminogramConcentration, motility, morphology, vitality, viability, count, semen volume, Progressive motility, non-Progressive motility (WHO parameters) - Secondary outcomes: Reproductive hormones, testosterone, MAR outcomes, live-birth, fertilization, implantation, and clinical pregnancy, sperm DNA fragmentation | - Genetics - Epigenetics - Proteomics |
| **Study design** | - Observational study - Clinical trial - Comparative study - Cross-sectional study | - Meta-analysis |
| **Article type** | - Original Research | - Review article - Systematic reviews - Case reports - Letters - Commentary articles |

**Supplemental Table 2.** PECOS (Population, Exposure, Comparison, Outcomes, Study design, Article type) for the inclusion and exclusion criteria. (MAR: Medically assisted reproductive).

**Supplemental Table 3.** Mediterranean dietary pattern components and scoring methodologies.

| **Trichopoulou Mediterranean diet (1)** | | | |
| --- | --- | --- | --- |
| **Components** | **Definition/examples** | **Dietary guideline** | **Scoring criteria** |
| Vegetables | All vegetables (excludes potatoes and French fries) | Encourage intake | 1 point for intake above median (servings/day); 0 for below |
| Legumes | Beans, lentils, chickpeas | Encourage intake | 1 point for intake above median (servings/day); 0 for below |
| Fruits and nuts | Fresh fruit, fruit juices, almonds, walnuts, etc. | Encourage intake | 1 point for intake above median (servings/day); 0 for below |
| Cereals | Both refined and whole grains | Encourage intake | 1 point for intake above median (servings/day); 0 for below |
| Meats | Red meat, processed meat, and organ meats products | Discourage intake | 1 point for intake below median (servings/day); 0 for above |
| Dairy | All types: full-fat, low-fat | Discourage intake | 1 point for intake below median (servings/day); 0 for above |
| Dietary fats | Ratio of monounsaturated to saturated fat | Encourage intake | 1 point for intake above median (servings/day); 0 for below |
| Fish and seafood | All fish and seafood | Encourage intake | 1 point for intake above median (servings/day); 0 for below |
| Alcohol | Wine, beer, liquor, etc. 100 mL = 12 g ethanol (g/day) | Moderate intake | **Males**: 1 point for 10–50 g/day; 0 for <10 or ≥50 g/day.  **Females**: 1 point for 5–25 g/day; 0 for <5 or ≥25 g/day. |
| **Alternate Mediterranean diet (2)** | | | |
| Vegetables | All vegetables (excludes potatoes and French fries) | Encourage intake | 1 point for intake above median (servings/day); 0 for below |
| Legumes | Beans, lentils, chickpeas | Encourage intake | 1 point for intake above median (servings/day); 0 for below |
| Fruits | Fresh fruit, fruit juices etc. | Encourage intake | 1 point for intake above median (servings/day); 0 for below |
| Nuts | Almonds, walnuts, etc. | Encourage intake | 1 point for intake above median (servings/day); 0 for below |
| Whole grains | Pasta, bread, rice, etc. made from whole grain | Encourage intake | 1 point for intake above median (servings/day); 0 for below |
| Meats | Red meat, processed meat, includes beef, deli meats, hot dogs, etc. (excludes chicken) | Discourage intake | 1 point for intake below median (servings/day); 0 for above |
| Dietary fats | Ratio of monounsaturated to saturated fat | Encourage intake | 1 point for intake above median (servings/day); 0 for below |
| Fish and seafood | All fish and seafood | Encourage intake | 1 point for intake above median (servings/day); 0 for below |
| Alcohol | Wine, beer, liquor (100 mL ≈ 12 g ethanol) | Moderate intake | **Males**: 15–25 g/day; Females: 5–15 g/day.  **Females**: <15 or ≥25 g/day; Females: <5 or ≥15 g/day. |
| **Panagiotakos Mediterranean diet (3)** | | | |
| Vegetables | All vegetables (excludes potatoes and French fries) | Encourage intake | 5 points: >33 servings/week  0 points: No vegetables |
| Legumes | Beans, lentils, chickpeas | Encourage intake | 5 points: >6 servings/week  0 points: No legumes |
| Fruits | Fresh fruit, fruit juices etc. | Encourage intake | 5 points: >22 servings/week  0 points: No fruits |
| Potatoes | Excludes French fries | Encourage intake | 5 points: >18 servings/week  0 points: No potatoes |
| Whole grains | Pasta, bread, etc. made from whole grain | Encourage intake | 5 points: >32 servings/week  0 points: No whole grains |
| Meats | Read meat and processed meat (e.g., hamburger, hot dog, deli meat, beef, etc., chicken not included) | Discourage intake | 5 points: ≤1 servings/week  0 points: >10 |
| Poultry | Chicken | Discourage intake | 5 points: ≤3 servings/week  0 points: >10 |
| Dairy product (full fat) | Full-fat cheese, yogurt, milk, etc. | Discourage intake | 5 points: ≤10 servings/week  0 points: >30 |
| Fish and seafood | All fish and seafood | Encourage intake | 5 points: >6 servings/week  0 points: No fish |
| Olive oil | Use of olive oil in cooking (times/ week) | Encourage intake | 5 points: 7 times/week  0 points: Never |
| Alcohol | Wine, beer, liquor (100 mL ≈ 12 g ethanol) | Moderate intake | 5 points: <300 mL/day  0 points: >700 mL/day or no alcohol |
| **Mediterranean Diet Adherence Screener (4)** | | | |
| Olive oil as main fat | Principal source of fat for cooking | Encouraged | 1 point: Yes (main source)  0 point: No |
| Olive oil consumption | Including that used in frying, salads, meals eaten away from home, etc | Encouraged | 1 point: ≥4 tbsp/day  0 point: <4 tbsp/day |
| Vegetable consumption | Side servings as 1/2 point; a full serving is 200 g | Encouraged | 1 point: ≥2 servings/day  0 point: <2 servings/day |
| Fruit consumption | Pieces of fruit (including fresh-squeezed juice) | Encouraged | 1 point: ≥3 servings/day  0 point: <3 servings/day |
| Meat | Sausages, red meat, or hamburger, full serving is 100–150 g | Discouraged | 1 point: <1 serving/day  0 point: ≥1 serving/day |
| Dairy | Butter, margarine, or cream | Discouraged | 1 point: <1 serving/day  0 point: ≥1 serving/day |
| Sugary beverages | Sugar-sweetened beverages or carbonated | Discouraged | 1 point: <1 serving/day  0 point: ≥1 serving/day |
| Wine | 1 glass/day (100–150 mL) | Encouraged | 1 point: Yes (1 glass/day)  0 point: No |
| Legumes | Pulses 150 g | Encouraged | 1 point: ≥3 servings/week  0 point: <3 servings/week |
| Fish or seafood | 100–150 g of fish, 4–5 pieces or 200 g of seafood | Encouraged | 1 point: ≥3 servings/week  0 point: <3 servings/week |
| Pastries/sweets | Commercial (not homemade) pastry such as cookies or cake per week | Discouraged | 1 point: <3 servings/week  0 point: ≥3 servings/week |
| Nuts | 1 serving = 30 g | Encouraged | 1 point: ≥3 servings/week  0 point: <3 servings/week |
| White meat | Rabbit, chicken or turkey | Encouraged | 1 point: Yes (if choosing white meat)  0 point: No |
| Sofrito consumption | Boiled vegetables, pasta, rice, or other dishes with a sauce of tomato, garlic, onion, or leeks sautéed in olive oil | Encouraged | 1 point: ≥2 servings/week  0 point: <2 servings/week |
| **Relative Mediterranean diet score (5)** | | | |
| Scoring for these 6 components T1: lowest tertile, T2: medium tertile, T3: highest tertile | | | |
| Fruits and nuts | Fresh fruits and nuts, excludes fruit juices | Encouraged | 0 points: T1  1 point: T2  2 points: T3 |
| Vegetables | All vegetables (excludes potatoes) | Encouraged | 0 points: T1  1 point: T2  2 points: T3 |
| Legumes | Beans, lentils, chickpeas, etc. | Encouraged | 0 points: T1  1 point: T2  2 points: T3 |
| Cereals | Whole-grain and refined flour, rice, pasta, other grains, and bread. | Encouraged | 0 points: T1  1 point: T2  2 points: T3 |
| Fish and seafood | All fish and seafood (excludes preserved and processed fish) | Encouraged | 0 points: T1  1 point: T2  2 points: T3 |
| Olive oil | Primary fat used; scored based on tertiles | Encouraged | 0 points: T1  1 point: T2  2 points: T3 |
| Scoring was reversed for these two food item groups, T1: highest tertile, T2: medium tertile, T3: lowest tertile | | | |
| Meat | Red meat and processed meat | Discouraged | 0 points: T1  1 point: T2  2 points: T3 |
| Dairy | Low-fat and high-fat milk, yogurt, cheese (including fresh cheese), cream desserts, and dairy and non-dairy creams | Discouraged | 0 points: T1  1 point: T2  2 points: T3 |
| Beneficial in moderation so, was scored as a dichotomous variable, either 0 or 2 | | | |
| Alcohol | Moderate intake: 5–25 g/day (women), 10–50 g/day (men) | Moderate intake | 0 points: Within range  2 points: Not in range |

**Abbreviations**: g, grams; mL, Milliliter; T, tertile.

**Supplemental Table 4**. Sensitivity analysis by systematic exclusion of one study at a time (sorted by author).

| **Removal of** | **Mean differences (MD)** | **95% CI** | **I² (%)** | **Comment** |
| --- | --- | --- | --- | --- |
| **Semen volume, mL** | | | | |
| **Overall** | 0.08 | -0.09, 0.25 | 53 |  |
| Davila-Cordova *et al*. 2024 MEDAS | 0.04 | -0.14, 0.22 | 53 | **-** |
| Karayiannis *et al*. 2017 PMD | 0.07 | -0.13, 0.28 | 59 | **-** |
| Palani *et al*. 2024 AMD | 0.04 | -0.15, 0.23 | 55 | - |
| Ricci *et al*. 2019 TMD | 0.05 | -0.18, 0.28 | 51 | **-** |
| Salas-Huetos *et al*. 2019 TMD | 0.07 | -0.11, 0.26 | 59 | **-** |
| Salas-Huetos *et al*. 2022 AMD | 0.10 | -0.10, 0.29 | 56 | **-** |
| Salas-Huetos *et al*. 2022 PMD | 0.13 | -0.03, 0.28 | 40 | Heterogeneity explained; changed from substantial to moderate. |
| Salas-Huetos *et al*. 2022 TMD | 0.13 | -0.03, 0.29 | 38 | Heterogeneity explained; changed from substantial to moderate. |
| **Sperm count, M spz.** | | | | |
| **Overall** | 24.37 | 1.30, 47.44 | 89 |  |
| Cutillas-Tolin *et al*. 2019 rMED | 24.86 | -0.10, 49.81 | 90 | Influential study; MD becomes non-significant. |
| Davila-Cordova *et al*. 2024 MEDAS | 20.33 | -3.69, 44.35 | 90 | Influential study; MD becomes non-significant. |
| Efrat *et al*. 2018 AMD | 27.94 | 2.23, 53.66 | 90 | - |
| Karayiannis *et al*. 2017 PMD | 25.42 | -0.84, 51.67 | 90 | Influential study; MD becomes non-significant. |
| Palani *et al*. 2024 AMD | 8.84 | -3.62, 21.30 | 56 | Influential study; MD changed more than 20%. Heterogeneity explained; changed from considerable to substantial. |
| Ricci *et al*. 2019 TMD | 26.17 | -6.81, 59.15 | 90 | Influential study; MD becomes non-significant. |
| Salas-Huetos *et al*. 2019 TMD | 27.97 | 2.97, 52.97 | 90 | - |
| Salas-Huetos *et al*. 2022 AMD | 27.07 | 1.20, 52.94 | 90 | - |
| Salas-Huetos *et al*. 2022 PMD | 28.58 | 3.48, 53.68 | 90 | - |
| Salas-Huetos *et al*. 2022 TMD | 28.45 | 3.33, 53.57 | 90 | - |
| **Sperm concentration, M spz./mL** | | | | |
| **Overall** | 7.41 | -1.26, 16.09 | 89 |  |
| Cutillas-Tolin *et al*. 2019 rMED | 8.71 | -0.42, 17.85 | 90 | - |
| Davila-Cordova *et al*. 2024 MEDAS | 6.27 | -3.01, 15.55 | 90 | - |
| Efrat *et al*. 2018 AMD | 8.23 | -1.46, 17.92 | 90 | **-** |
| Karayiannis *et al*. 2017 PMD | 7.34 | -2.60, 17.28 | 90 | - |
| Palani *et al*. 2024 AMD | 3.93 | -0.02, 7.89 | 35 | Influential study; MD changed more than 20%. Heterogeneity explained; changed from considerable to moderate. |
| Ricci *et al*. 2019 TMD | 7.23 | -5.08, 19.54 | 90 | **-** |
| Salas-Huetos *et al*. 2019 TMD | 8.65 | -0.65, 17.95 | 90 | - |
| Salas-Huetos *et al*. 2022 AMD | 8.14 | -1.35, 17.63 | 90 | **-** |
| Salas-Huetos *et al*. 2022 PMD | 8.14 | -1.28, 17.56 | 90 | **-** |
| Salas-Huetos *et al*. 2022 TMD | 8.02 | -1.47, 17.52 | 90 | **-** |
| **Sperm total motility, %** | | | | |
| **Overall** | 8.81 | 2.26, 15.37 | 88 |  |
| Cutillas-Tolin *et al*. 2019 rMED | 10.03 | 2.73, 17.33 | 88 | - |
| Davila-Cordova *et al*. 2024 MEDAS | 9.29 | 1.66, 16.91 | 89 | - |
| Efrat *et al*. 2018 AMD | 9.63 | 2.12, 17.14 | 89 | **-** |
| Karayiannis *et al*. 2017 PMD | 8.64 | 1.27, 16.10 | 89 | **-** |
| Palani *et al*. 2024 AMD | 5.06 | 1.54, 8.57 | 54 | Heterogeneity explained; changed from considerable to substantial. |
| Salas-Huetos *et al*. 2019 TMD | 7.78 | 0.92, 14.64 | 88 | **-** |
| Salas-Huetos *et al*. 2022 AMD | 9.30 | 1.88, 16.73 | 89 | **-** |
| Salas-Huetos *et al*. 2022 PMD | 9.94 | 2.81, 17.07 | 89 | **-** |
| Salas-Huetos *et al*. 2022 TMD | 9.52 | 2.24, 16.79 | 89 | **-** |
| **Sperm progressive motility, %** | | | | |
| **Overall** | 7.49 | 1.47, 13.50 | 86 |  |
| Davila-Cordova *et al*. 2024 MEDAS | 7.96 | 0.85, 15.07 | 88 | - |
| Karayiannis *et al*. 2017 PMD | 7.88 | 0.67, 15.08 | 88 | - |
| Palani *et al.* 2024 AMD | 3.32 | 0.73, 5.91 | 22 | Influential study; MD changed more than 20%. Heterogeneity explained; changed from considerable to moderate. |
| Salas-Huetos *et al*. 2019 TMD | 6.90 | 0.44, 13.35 | 88 | - |
| Salas-Huetos *et al*. 2022 AMD | 8.57 | 1.23, 15.92 | 88 | - |
| Salas-Huetos *et al*. 2022 PMD | 8.96 | 2.03, 15.88 | 87 | - |
| Salas-Huetos *et al*. 2022 TMD | 8.58 | 1.42, 15.74 | 88 | - |
| **Sperm normal morphology, %** | | | | |
| **Overall** | 1.02 | 0.21, 1.82 | 77 |  |
| Cutillas-Tolin *et al*. 2019 rMED | 1.05 | 0.20, 1.89 | 79 | - |
| Davila-Cordova *et al*. 2024 MEDAS | 0.96 | 0.11, 1.81 | 80 | - |
| Efrat *et al*. 2018 AMD | 1.15 | 0.33, 1.97 | 78 | **-** |
| Karayiannis *et al*. 2017 PMD | 1.13 | 0.28, 1.99 | 74 | **-** |
| Palani *et al*. 2024 AMD | 0.73 | 0.32, 1.14 | 0 | Influential study; MD changed more than 20%. Heterogeneity explained; changed from considerable to moderate. |
| Salas-Huetos *et al*. 2019 TMD | 1.13 | 0.27, 1.98 | 77 | **-** |
| Salas-Huetos *et al*. 2022 AMD | 1.01 | 0.09, 1.92 | 79 | **-** |
| Salas-Huetos *et al*. 2022 PMD | 0.95 | 0.03, 1.88 | 80 | **-** |
| Salas-Huetos *et al*. 2022 TMD | 1.00 | 0.05, 1.95 | 79 | **-** |

Summary MD from Random effects recalculated after the systematical removal of one study at a time. We considered an influential study as the one whose exclusion changed the significance, direction or magnitude (by >20%) of the pooled MD or changed the magnitude of the heterogeneity (e.g. considerable heterogeneity to substantial). We assume that I^2^ values indicate moderate heterogeneity for values <50%, substantial heterogeneity if the values are between ≥50 and <75%, and considerable heterogeneity for ≥75%.

**Abbreviations**: aMED, alternate Mediterranean Diet score; AMD, Alternate Mediterranean diet; CI, confidence interval; I², Heterogeneity; MD, mean differences; MEDAS, Mediterranean Diet Adherence Screener; MedDiet, Mediterranean Diet; M, million; PBD, Plant-based diet score; PMD, Panagiotakos Mediterranean diet; rMED, relative Mediterranean diet score; spz., spermatozoa.; TMD, Trichopoulou Mediterranean diet.


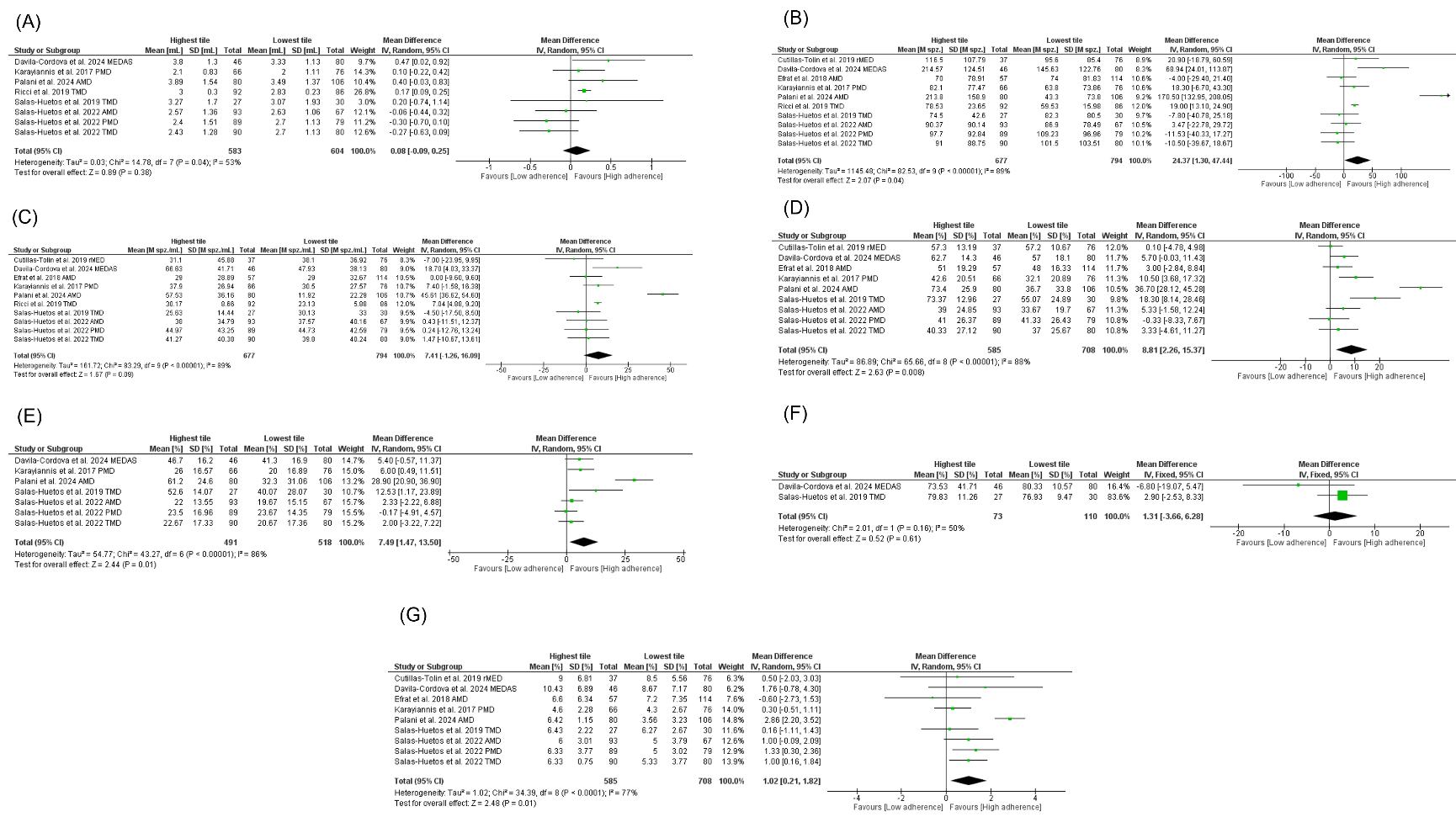
**Supplemental Figure 1.** Forest plot of mean differences (MD) and 95% confidence intervals (CI) for studies evaluating the association between adherence to Total MedDiet and (A) Semen volume [mL], (B) Sperm count [M spz.], (C) Sperm concentration [M spz./mL], (D) Sperm total motility [%], (E) Sperm progressive motility [%], (F) Sperm vitality [%], (G) Sperm normal morphology [%].

Green squares boxes for each study indicate the MD, the size of the boxes indicates the weight of the study, and the horizontal lines indicate the 95% CI. The bold and diamond shape data represent the pooled MD and 95% CI. Overall estimates were obtained using Random-effect models for more than 5 studies involved.


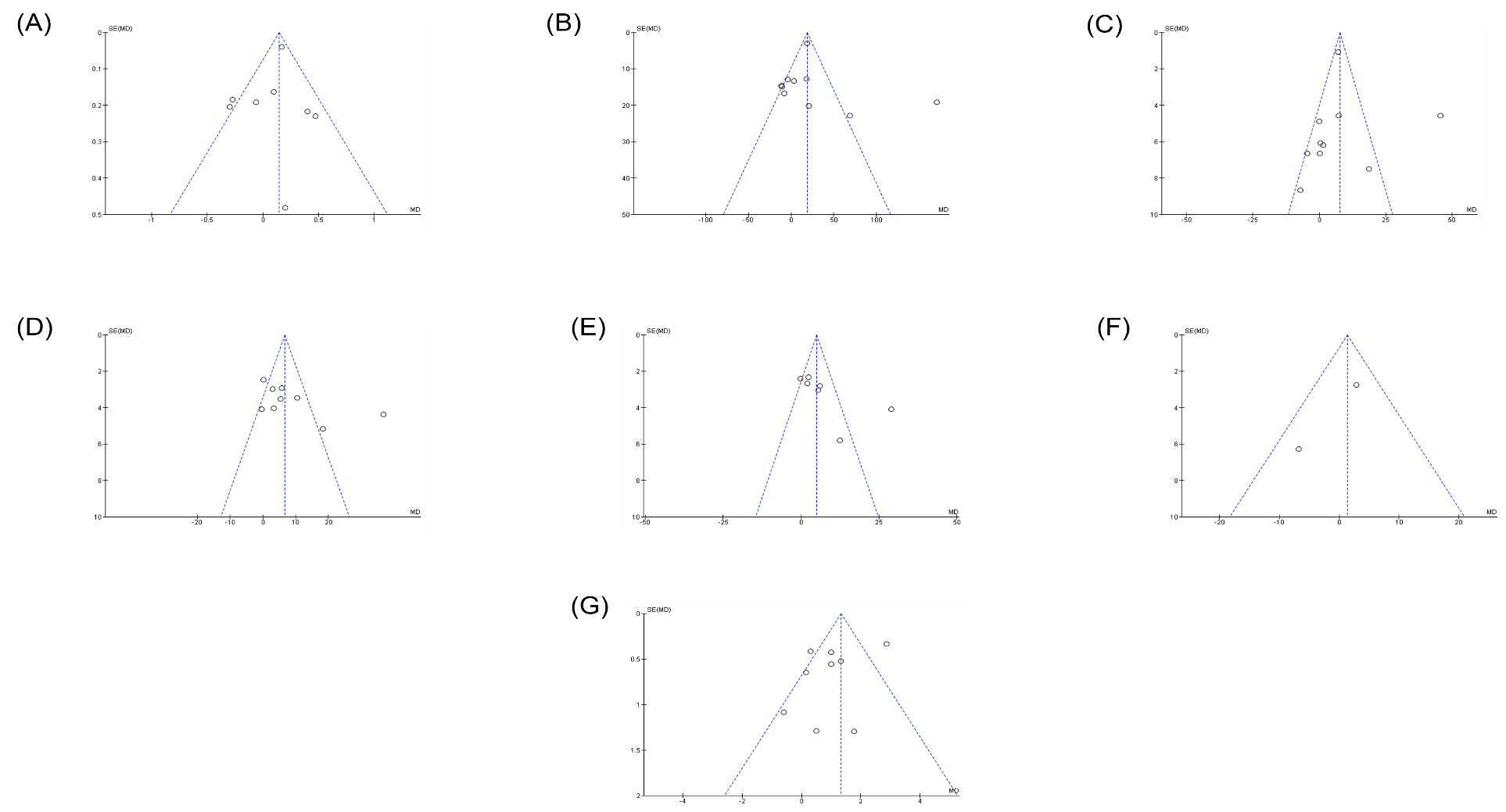
**Supplemental Figure 2.** Funnel plot of the meta-analysis plotted with standard error (SE) on the y-axis and mean difference (MD) on the x-axis, evaluating the association between adherence to Total MedDiet and (A) Semen volume [mL], (B) Sperm count [M spz.], (C) Sperm concentration [M spz./mL], (D) Sperm total motility [%], (E) Sperm progressive motility [%], (F) Sperm vitality [%], (G) Sperm normal morphology [%].

The triangle represents the region where 95% of the data points should lie in the absence of publication bias. The vertical line represents the average standardized mean difference found in the meta-analysis.


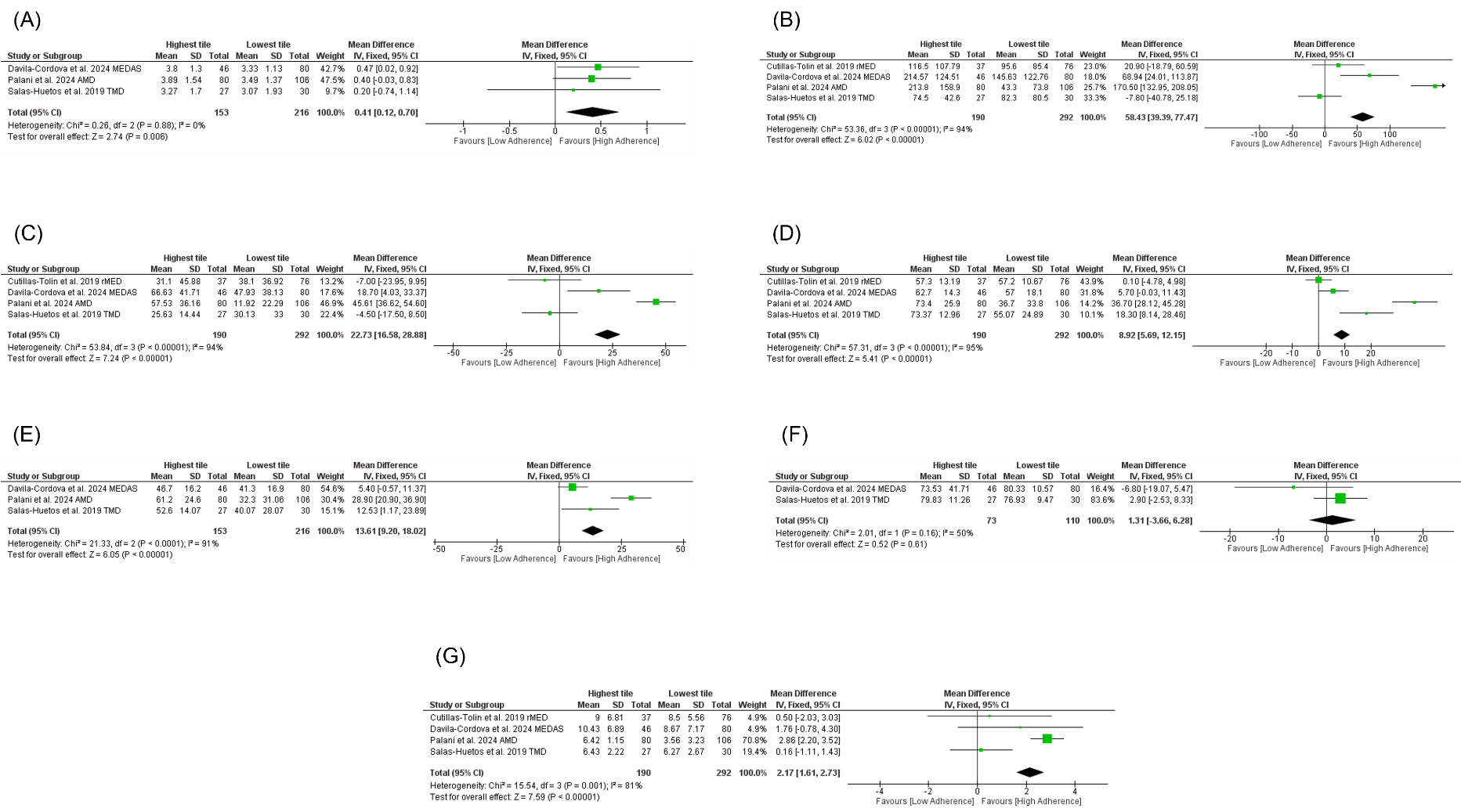
**Supplemental Figure 3.** Forest plot of mean differences (MD) and 95% confidence intervals (CI) for studies evaluating the association between adherence to Total MedDiet and (A) Semen volume [mL], (B) Sperm count [M spz.], (C) Sperm concentration [M spz./mL], (D) Sperm total motility [%], (E) Sperm progressive motility [%], (F) Sperm vitality [%], (G) Sperm normal morphology [%] in the participants from Healthy population.

Green squares boxes for each study indicate the MD, the size of the boxes indicates the weight of the study, and the horizontal lines indicate the 95% CI. The bold and diamond shape data represent the pooled MD and 95% CI. Overall estimates were obtained using Fixed-effect models for less than 5 studies involved.

**
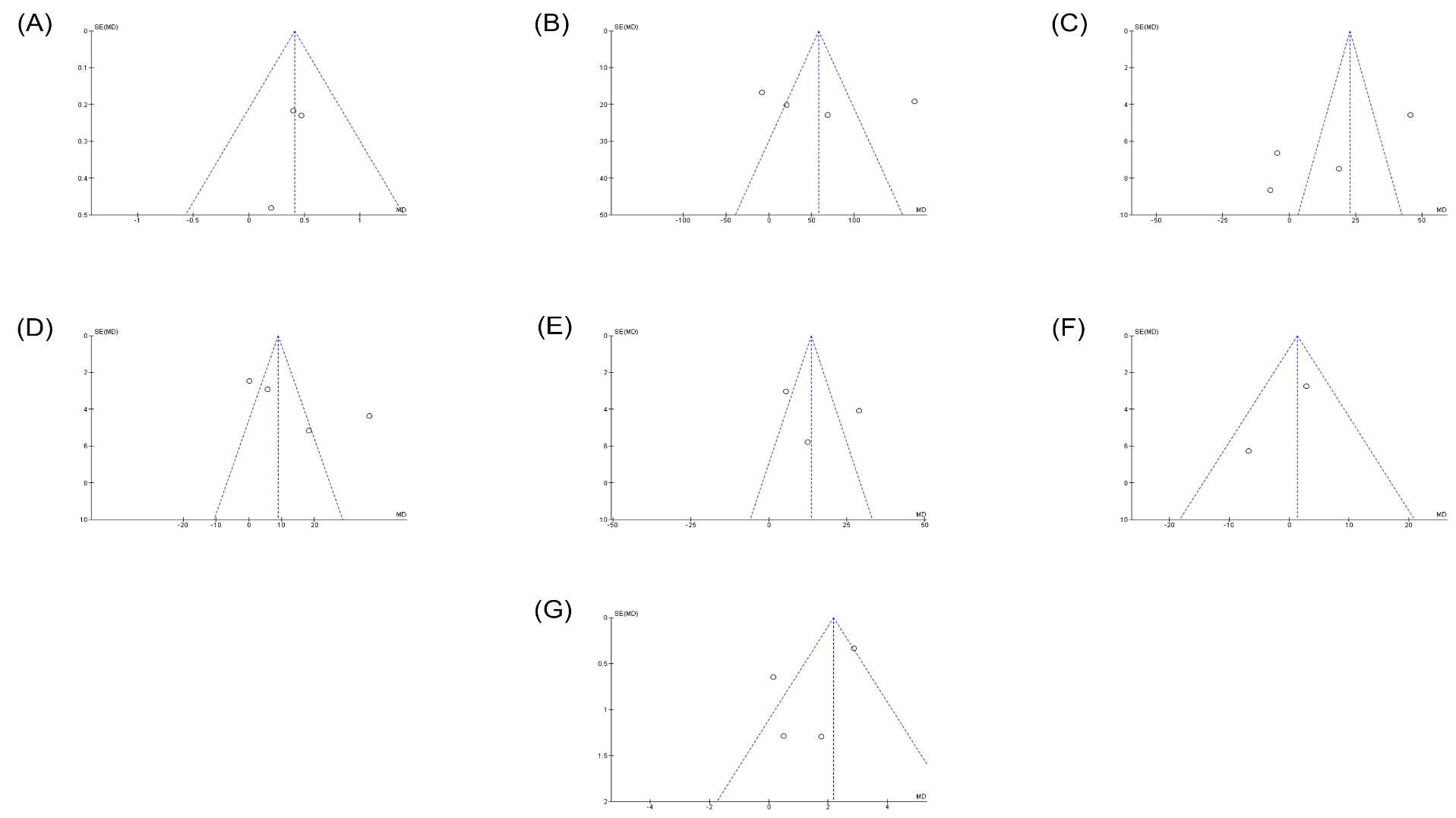
Supplemental Figure 4.** Funnel plot of the meta-analysis plotted with standard error (SE) on the y-axis and mean difference (MD) on the x-axis, evaluating the association between adherence to Total MedDiet and (A) Semen volume [mL], (B) Sperm count [M spz.], (C) Sperm concentration [M spz./mL], (D) Sperm total motility [%], (E) Sperm progressive motility [%], (F) Sperm vitality [%], (G) Sperm normal morphology [%] in the participants from Healthy population.

The triangle represents the region where 95% of the data points should lie in the absence of publication bias. The vertical line represents the average standardized mean difference found in the meta-analysis.

**Supplemental Figure 5.** Forest plot of mean differences (MD) and 95% confidence intervals (CI) for studies evaluating the association between adherence to Total MedDiet pattern and (A) Semen volume [mL], (B) Sperm count [M spz.], (C) Sperm concentration [M spz./mL], (D) Sperm total motility [%], (E) Sperm progressive motility [%], (F) Sperm normal morphology [%] in participants recruited from fertility clinics.


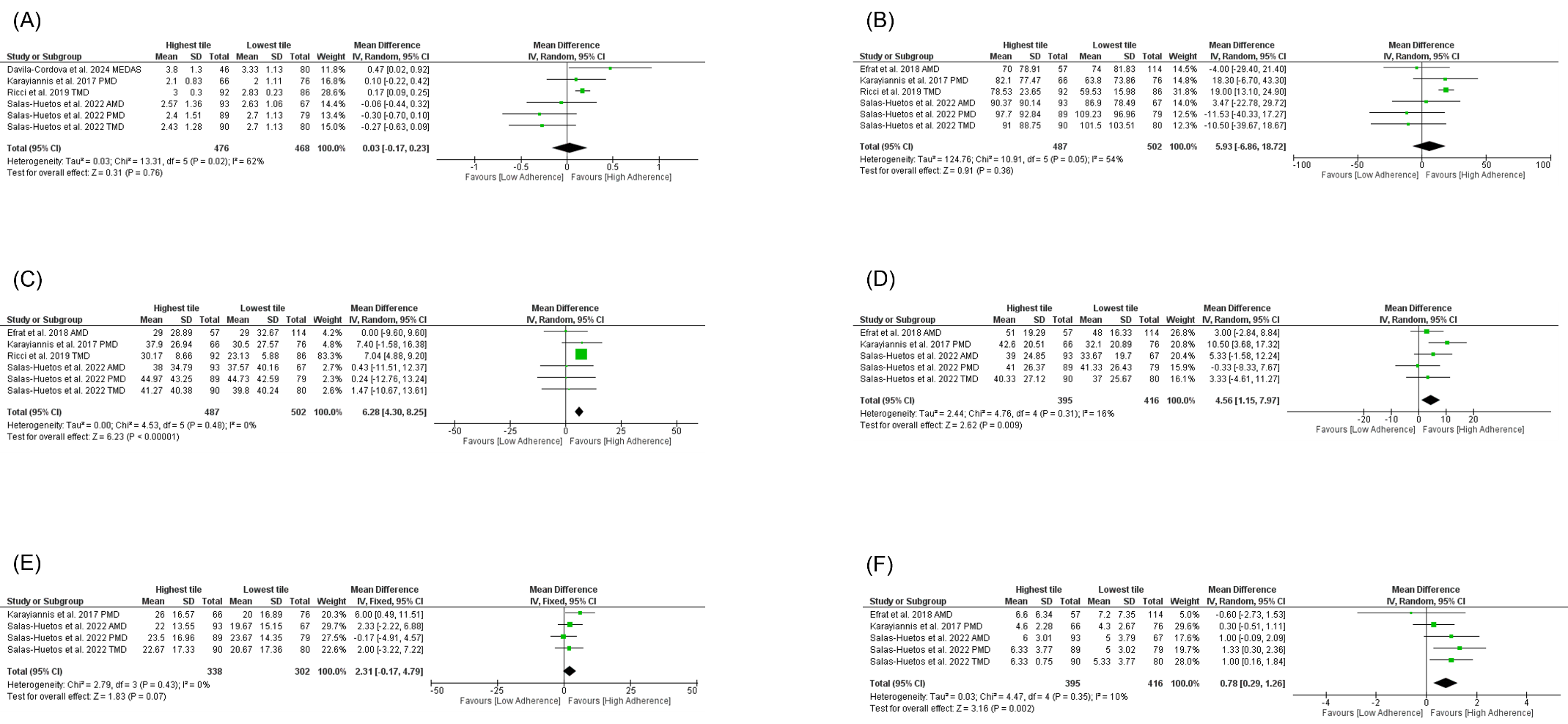


Green squares boxes for each study indicate the MD, the size of the boxes indicates the weight of the study, and the horizontal lines indicate the 95% CI. The bold and diamond shape data represent the pooled MD and 95% CI. Overall estimates were obtained using Fixed-effect models for less than 5 studies.

**Supplemental Figure 6.** Funnel plot of the meta-analysis plotted with standard error (SE) on the y-axis and mean difference (MD) on the x-axis, evaluating the association between adherence to Total MedDiet pattern and (A) Semen volume [mL], (B) Sperm count [M spz.], (C) Sperm concentration [M spz./mL], (D) Sperm total motility [%], (E) Sperm progressive motility [%], (F) Sperm normal morphology [%] in participants recruited from fertility clinics.

**
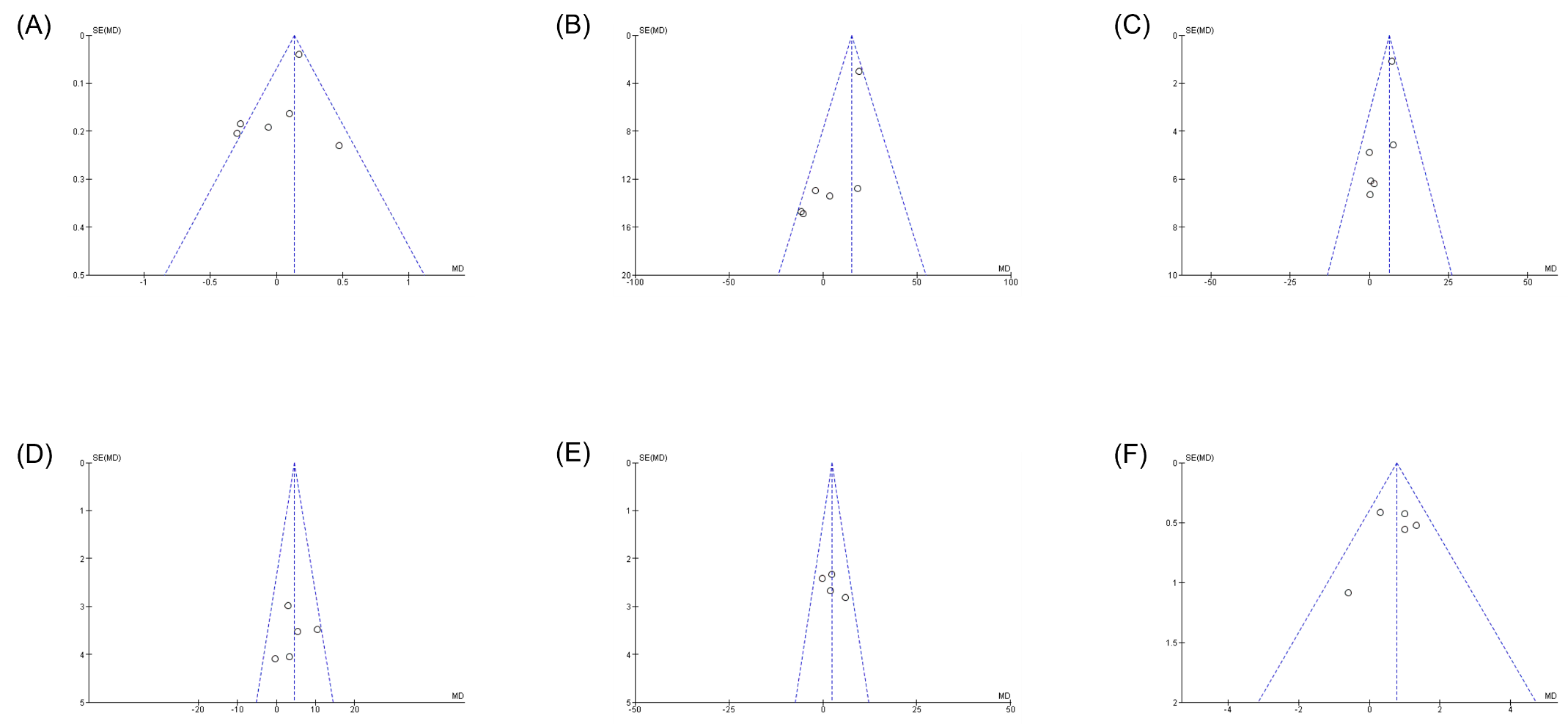
**

The triangle represents the region where 95% of the data points should lie in the absence of publication bias. The vertical line represents the average standardized mean difference found in the meta-analysis.

**Supplemental Figure 7.** Forest plot of mean differences (MD) and 95% confidence intervals (CI) for studies evaluating the association between adherence to TMD diet pattern and (A) Semen volume [mL], (B) Sperm count [M spz.], (C) Sperm concentration [M spz./mL], (D) Sperm total motility [%], (E) Sperm progressive motility [%], (F) Sperm normal morphology [%].


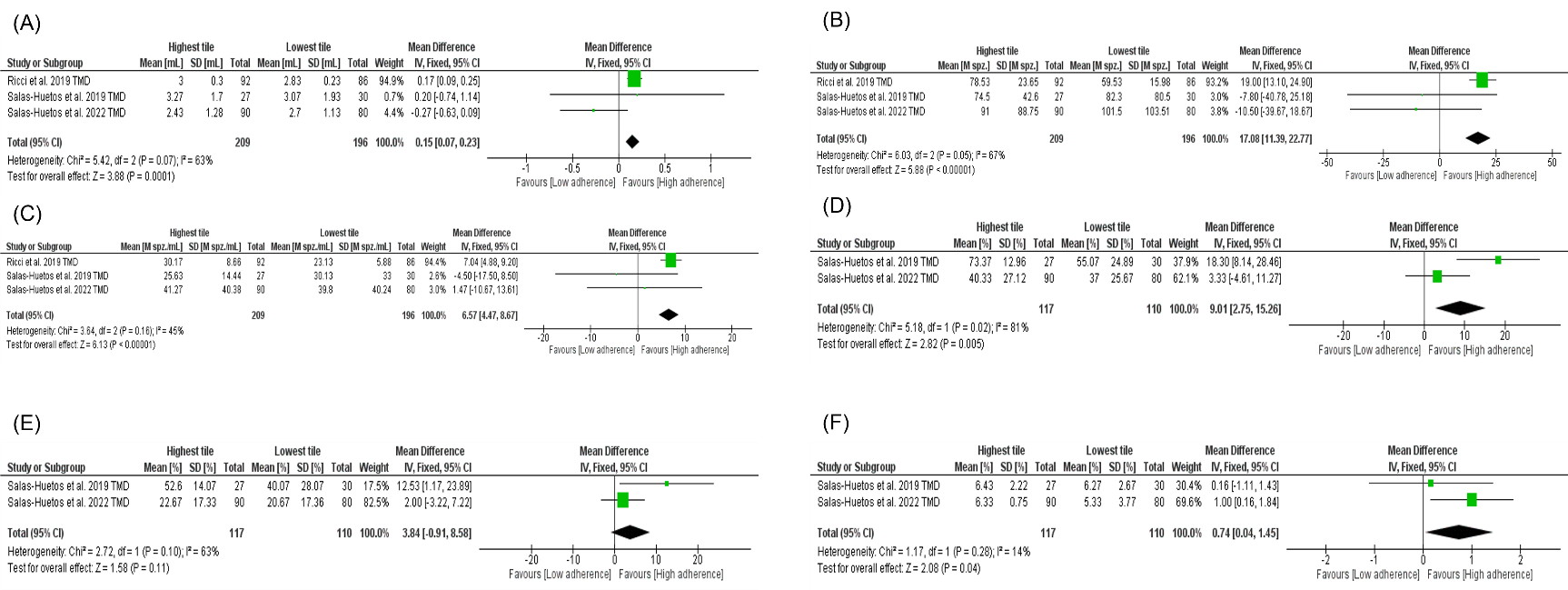


Green squares boxes for each study indicate the MD, the size of the boxes indicates the weight of the study, and the horizontal lines indicate the 95% CI. The bold and diamond shape data represent the pooled MD and 95% CI. Overall estimates were obtained using Fixed-effect models for less than 5 studies.

**Supplemental Figure 8.** Forest plot of mean differences (MD) and 95% confidence intervals (CI) for studies evaluating the association between adherence to AMD diet pattern and (A) Semen volume [mL], (B) Sperm count [M spz.], (C) Sperm concentration [M spz./mL], (D) Sperm total motility [%], (E) Sperm progressive motility [%], (F) Sperm normal morphology [%].


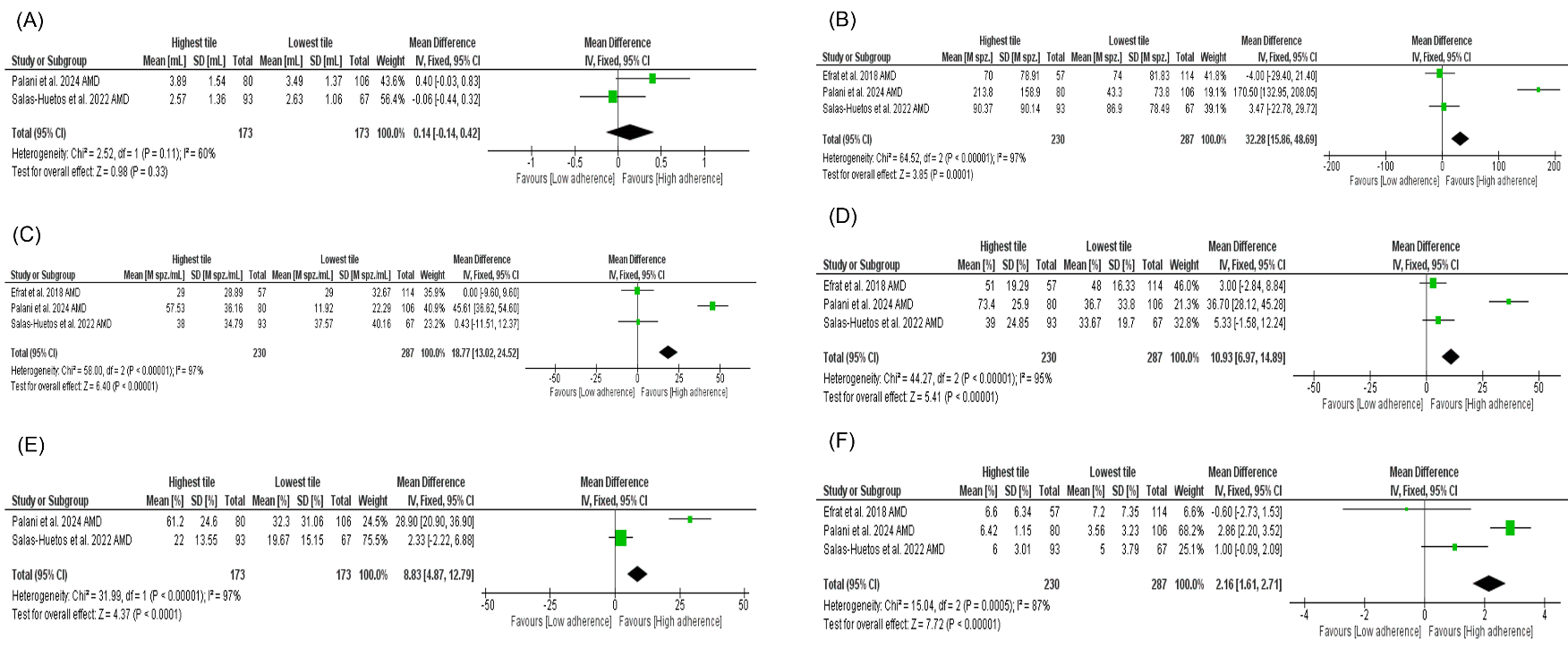


Green squares boxes for each study indicate the MD, the size of the boxes indicates the weight of the study, and the horizontal lines indicate the 95% CI. The bold and diamond shape data represent the pooled MD and 95% CI. Overall estimates were obtained using Fixed-effect models for less than 5 studies.

**Supplemental Figure 9.** Forest plot of mean differences (MD) and 95% confidence intervals (CI) for studies evaluating the association between adherence to PMD diet pattern and (A) Semen volume [mL], (B) Sperm count [M spz.], (C) Sperm concentration [M spz./mL], (D) Sperm total motility [%], (E) Sperm progressive motility [%], (F) Sperm normal morphology [%].

**
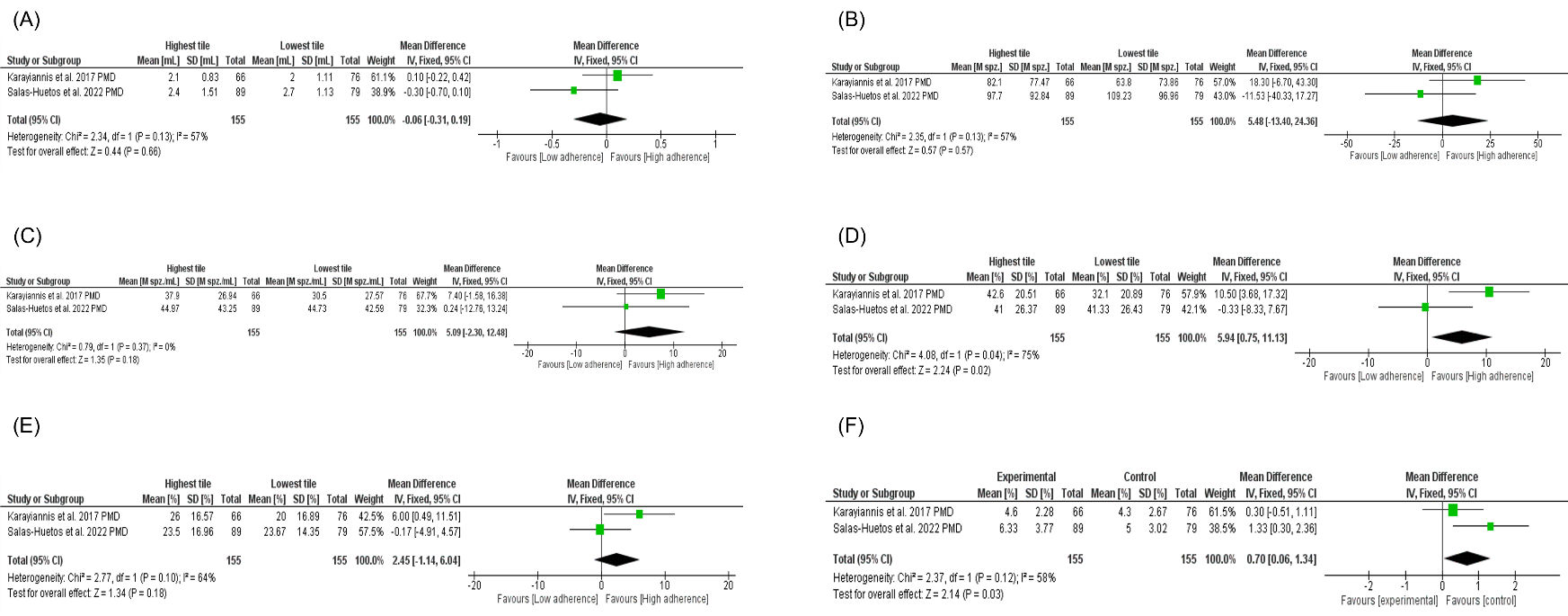
**

Green squares boxes for each study indicate the MD, the size of the boxes indicates the weight of the study, and the horizontal lines indicate the 95% CI. The bold and diamond shape data represent the pooled MD and 95% CI. Overall estimates were obtained using Fixed-effect models for less than 5 studies.

**References**

1. Trichopoulou A, Costacou T, Bamia C, Trichopoulos D. Adherence to a Mediterranean Diet and Survival in a Greek Population. N Engl J Med [Internet] 2003;26:2599–608.

2. Fung TT, McCullough ML, Newby P, Manson JE, Meigs JB, Rifai N, Willett WC, Hu FB. Diet-quality scores and plasma concentrations of markers of inflammation and endothelial dysfunction 1-3. Am J Clin Nutr [Internet] 2005;82:163–73.

3. Panagiotakos DB, Pitsavos C, Arvaniti F, Stefanadis C. Adherence to the Mediterranean food pattern predicts the prevalence of hypertension, hypercholesterolemia, diabetes and obesity, among healthy adults; the accuracy of the MedDietScore. Prev Med (Baltim) 2007;44:335–40.

4. Schröder H, Fitó M, Estruch R, Martínez-González MA, Corella D, Salas-Salvadó J, Lamuela-Raventós R, Ros E, Salaverría I, Fiol M, et al. A Short screener is valid for assessing mediterranean diet adherence among older spanish men and women. Journal of Nutrition 2011;141:1140–5.

5. Buckland G, González CA, Agudo A, Vilardell M, Berenguer A, Amiano P, Ardanaz E, Arriola L, Barricarte A, Basterretxea M, et al. Adherence to the mediterranean diet and risk of coronary heart disease in the spanish EPIC cohort study. Am J Epidemiol 2009;170:1518–29.
